# Lead (Pb) Isotope Baselines for Studies of Ancient Human Migration and Trade in the Maya Region

**DOI:** 10.1371/journal.pone.0164871

**Published:** 2016-11-02

**Authors:** Ashley E. Sharpe, George D. Kamenov, Adrian Gilli, David A. Hodell, Kitty F. Emery, Mark Brenner, John Krigbaum

**Affiliations:** 1 Department of Anthropology, University of Florida, Gainesville, Florida, United States of America; 2 Environmental Archaeology Program, Florida Museum of Natural History, University of Florida, Gainesville, Florida, United States of America; 3 Department of Geological Sciences, University of Florida, Gainesville, Florida, United States of America; 4 Department of Earth Sciences, Eidgenössische Technische Hochschule Zürich, Zürich, Switzerland; 5 Department of Earth Sciences, University of Cambridge, Cambridge, United Kingdom; New York State Museum, UNITED STATES

## Abstract

We examined the potential use of lead (Pb) isotopes to source archaeological materials from the Maya region of Mesoamerica. The main objectives were to determine if: 1) geologic terrains throughout the Maya area exhibit distinct lead isotope ratios (^206^Pb/^204^Pb, ^207^Pb/^204^Pb, and ^208^Pb/^204^Pb), and 2) a combination of lead and strontium ratios can enhance sourcing procedures in the Mesoamerica region. We analyzed 60 rock samples for lead isotope ratios and a representative subset of samples for lead, uranium, and thorium concentrations across the Maya region, including the Northern Lowlands of the Mexican Yucatan Peninsula, the Southern Lowlands of Guatemala and Belize, the Volcanic Highlands, the Belizean Maya Mountains, and the Metamorphic Province/Motagua Valley. Although there is some overlap within certain sub-regions, particularly the geologically diverse Metamorphic Province, lead isotopes can be used to distinguish between the Northern Lowlands, the Southern Lowlands, and the Volcanic Highlands. The distinct lead isotope ratios in the sub-regions are related to the geology of the Maya area, exhibiting a general trend in the lowlands of geologically younger rocks in the north to older rocks in the south, and Cenozoic volcanic rocks in the southern highlands. Combined with other sourcing techniques such as strontium (^87^Sr/^86^Sr) and oxygen (δ^18^O), a regional baseline for lead isotope ratios can contribute to the development of lead isoscapes in the Maya area, and may help to distinguish among geographic sub-regions at a finer scale than has been previously possible. These isotope baselines will provide archaeologists with an additional tool to track the origin and movement of ancient humans and artifacts across this important region.

## Introduction

Recent progress in the application of geochemical sourcing techniques using archaeological materials enables both new and improved methods to investigate past movements of people and objects across landscapes [1–6]. Just as radiocarbon dating allows archaeologists to track processes through time in the archaeological record, radiogenic isotopes of elements such as strontium and lead and light isotopes of oxygen provide a means to examine spatial dimensions of materials recovered in the archaeological record.

**Fig 1:**
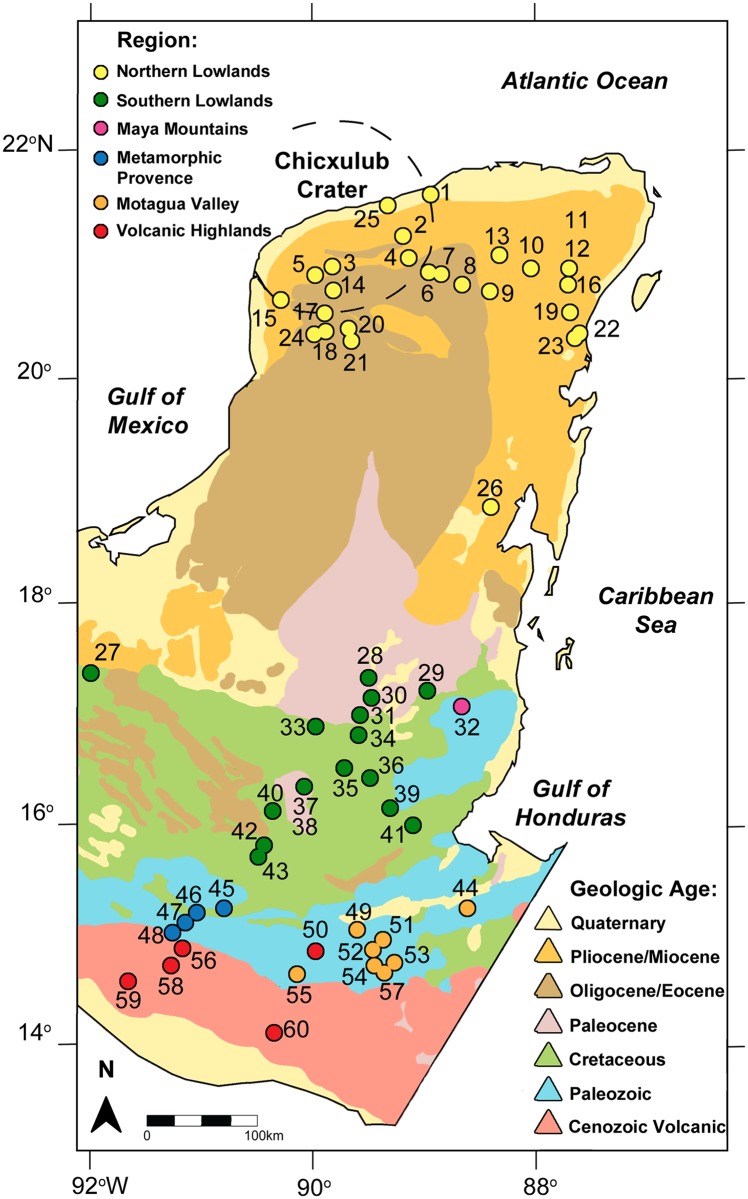
Geological map of the Mesoamerican region. Samples from this study are denoted by colored circles. Sample numbers correlate with the samples described in Table 1. The Chicxulub crater basin is denoted by dashed lines. Map adapted from the U.S. Geological Survey Geologic Map of North America (public domain, http://ngmdb.usgs.gov/gmna/).

This study provides a requisite first step to evaluate spatial differences in lead isotope ratios across the Maya region of Mesoamerica (Fig 1). The ancient Maya political and economic system relied on movements of people and products, including animals, ceramic vessels, chert, and obsidian [7–12]. Lead can be used in conjunction with other more established techniques, such as strontium and oxygen isotope analysis, to investigate the movement of humans and resources in the past. Combined, multi-isotope approaches that include lead isotopes could facilitate greater discrimination in tracing humans and non-human faunal resources with greater accuracy, and increase the resolution with which we can track past migration and trade routes. The complex geology of the Maya region, with its long and rich history of human occupation, makes it an excellent location to explore the application of lead sourcing using archaeological materials.

Isotopes of an element can be used to differentiate culturally connected regions at different spatial scales, depending upon the particular research question addressed. In Mesoamerica, geochemical studies on archaeological human and animal remains have primarily relied on oxygen and strontium isotopes. Oxygen (δ^18^O) in archaeological remains can be used to distinguish among geographic areas, but is ineffective for detailed sourcing studies as a consequence of myriad factors including precipitation, distance from an ocean, temperature, and elevation [13–16]. Strontium, using the ^87^Sr/^86^Sr ratio for isotopic sourcing studies, can distinguish among broad regions within Mesoamerica [17], such as between the lowlands and highlands of Guatemala and southern Mexico. Strontium ratios have been used to track movements of ancient humans and animal resources [11, 12, 14, 16, 18–20]; however, strontium ratios in different areas of the Maya lowlands exhibit significant overlap.

In this study, we analyzed lead isotopes from geologic samples collected across the region, which was densely occupied between *ca*. 1000 BCE and CE 1000 by the ancient Maya, and is populated today by their descendants. Lead isotopes can be used to source archaeological cultural materials, such as ceramic components, glazes, metals, and stone, but are particularly useful in that they can be used to source human and animal tooth enamel (and potentially bone), enabling studies of human mobility and trade of faunal materials. A growing number of studies have included isotope ratios of lead as an additional geochemical sourcing technique [21–27]. In the Americas, lead has been tested in only a few studies, such as archaeological human remains from Peru [28], the southwestern United States [29, 30] and Canada [31], and for sourcing minerals and metals, such as glazes, turquoise, and copper in the exchange network that existed between the southwestern United States through Mesoamerica [32–35]. Lead isotope sourcing was applied in a study examining the possible Old World origin of individuals recovered from beneath a colonial-era church and associated burial ground in Campeche, Mexico [36], although the lead isotope results could only be used to distinguish between broad New and Old World categories because no Mesoamerican regional lead isotope baseline existed. Examining the degree of lead isotope variation across the geographically small, but geologically diverse Maya area, is an important first step to address questions pertaining to past human migration and trade in Mesoamerica.

This study builds on the previous investigation by Hodell et al. [17] that provided a baseline strontium (^87^Sr/^86^Sr) map for the Maya area, and which has since been expanded upon in subsequent studies [11, 12, 14, 16, 18–20, 37]. For this study, we analyzed a collection of geological samples from the Maya region to create a similar baseline for lead isotopes. The primary objectives of this investigation were to: 1) determine if the geologic terrains of the Maya area possess lead ratios that are sufficiently different to use for sourcing and migration studies, and 2) test whether lead ratios, when used in conjunction with strontium, can better define geographic sub-regions and thus provide a more detailed tool for human and cultural material sourcing.

## Lead Isotope Geochemistry

Lead has one non-radiogenic isotope, ^204^Pb, and three isotopes produced by radiogenic decay, ^206^Pb, ^207^Pb, and ^208^Pb. ^206^Pb and ^207^Pb are products of ^238^U and ^235^U decay, respectively, and ^208^Pb is a product of ^232^Th decay. The abundances of these four lead isotopes vary geographically, depending on the age of the bedrock, U/Pb and Th/Pb, and anthropogenic factors [38, 39]. Thus, lead isotopes in a region provide three independent ratios to compare for sourcing archaeological materials. Lead can be added to the roster of other isotopes used in Mesoamerican trade and migration studies, of which two, oxygen and strontium, are most commonly used.

Oxygen, strontium, and lead are incorporated adventitiously into the body of organisms, including the skeletons and tooth enamel of vertebrates, through inhalation and the consumption of food and water. Like strontium, lead substitutes for calcium in bones and teeth [40]. In contrast to strontium and oxygen, lead is incorporated in humans via direct exposure to soil or dust containing lead by breathing and ingesting, without the fractionation typical of lighter isotopes incorporated through the diet ([41] page 7), and can therefore be readily linked to the local bedrock geology ([39] and references therein). The lead isotope composition of the soil in each region will depend to some extent on the weathering regime. Physical weathering will deliver mostly unaltered bedrock components, with their heterogeneous lead isotope signatures, to the developing soil horizon. Extensive chemical weathering, on the other hand, results in the breakdown of many rock-forming minerals. This averages the soil lead isotope signal inherited from the bedrock. Because of the warm and humid climate in the Maya area, chemical weathering is the dominant process that controls incorporation of lead from the bedrock into local soils. Therefore, we propose that the bedrock-defined lead isotopes are representative for the soils and bioavailable lead as well. To test this hypothesis, as part of this study, we conducted a leaching experiment with several limestone samples to test whether the residual silicate fractions were isotopically similar to the more soluble carbonate fractions.

Both ancient and modern anthropogenic sources of lead (i.e. pollution) have been found to contaminate "natural" lead signatures. In Europe, lead produced as a byproduct of silver and lead mining by the ancient Greeks, Romans, and other groups, beginning ~3000 years ago, has been detected in Greenland ice cores [42] and human teeth [43, 44]. More recently, anthropogenic lead released into the environment as a consequence of mining and leaded gasoline use has even contaminated the uninhabited ice sheets of Antarctica [45]. Virtually all modern humans have some anthropogenic lead in their teeth [39].

The Mesoamerican region has seen a considerable rise in population, construction, and land development over the past several decades, increasing the potential for lead contamination. Fortunately, the Maya lowlands lacked substantial mining and metal smelting activity until the arrival of Spanish colonists in the 16th century. Relatively few instances of small copper artifacts such as bells have been reported in the southern Mesoamerican lowlands, having come from west and central Mexico during the Classic and Postclassic periods (*ca*. CE 500–1500) as rare luxury imports [33]. Smelting processes at these times were not as intensive as those that took place in the Old World and the Andes mountains of South America, and were confined to relatively few areas; as such, considerable lead contamination did not occur in Mesoamerica until recently. Nevertheless, these sources of modern lead contamination, in particular the widespread use of leaded gasoline in the near past, complicate the task of establishing lead isotope baselines. In contrast to the approach used to produce the strontium map for the region [17], we could not use modern plant and water samples to produce a lead isotope ratio baseline because the samples may be contaminated by modern lead pollution to varying extents. We instead used rock samples to obtain natural lead ratios reflective of pre-industrial times.

## Geology of the Maya Region

The geographic area covered in this study spans the Yucatan Peninsula in Mexico, south through Guatemala, and into Belize and western Honduras. A notable feature of the area is the gradual increase in the geological age of the bedrock from north to south in addition to Cenozoic volcanic rocks in the southernmost part (Fig 1 and [17]). The region can be separated into five geographically distinct sub-regions based on differences in the age and type of rock: 1) Northern Lowlands, 2) Southern Lowlands, 3) Volcanic Highlands and Pacific Coast, 4) Metamorphic Province, and 5) the Maya Mountains. Hodell et al. [17] carried out the first intensive strontium study in the area, which showed that strontium ratios varied among the sub-regions and could be used to track ancient migration and trade. Their findings were corroborated and elaborated upon by later strontium investigations [11, 12, 14, 16, 18, 19, 37, 46], which also added more detail to the variations between and within micro-regions such as the Motagua and Copan Valleys (hereafter referred to collectively as the Motagua Valley, the Copan River being a tributary of the Motagua River), which is a separate region within the generalized Metamorphic Province.

The Northern Lowlands are characterized by a flat carbonate platform of marine limestone that increases in age from north to south, although exposures of different rocks have been noted in different areas, such as Pliocene-Pleistocene deposits along the north coast, Miocene limestone across much of the northern region, and Eocene limestone in the north-central Yucatan [17, 37]. Strontium ratios for this area tend to range between 0.70775 and 0.70921, the latter reflecting the modern value for seawater and appearing most frequently near the coast and inside the Chicxulub impact crater (Fig 1).

The Northern Lowlands gradually give way to the Southern Lowlands in southern Mexico and northern Guatemala and Belize, an area of higher elevation than most of the Yucatan Peninsula. The limestone deposits of this region tend to be older, of Paleocene and Cretaceous age, with strontium ratios ranging from 0.70693 to 0.70845. One difficulty observed by Hodell et al. [17] and confirmed in later studies [11, 12, 16, 19] is that the Southern Lowlands, which encompass a large area and a number of important Maya sites, unfortunately do not exhibit a great deal of variation in their strontium ratios, making it difficult to identify migration and exchange between sites within the area. Oxygen isotopes have been used as an additional means of tracking movement in this area because δ^18^O can reflect differences in precipitation and the size and type of nearby water bodies (e.g. lakes, *aguadas*, and reservoirs); however, studies have found that humans exhibiting similar strontium ratios at a single site may have significantly different δ^18^O values [16], and that catchment sites themselves can exhibit significant δ^18^O variation throughout the year [15]. Given the older age of the underlying sedimentary rock (mostly limestone) in this region, compared to bedrock in the Northern Lowlands, we would expect lead isotope ratios from this area to be more radiogenic than the Yucatan rocks. Furthermore, the variable age of the sedimentary rocks (Fig 1) may be reflected in the lead isotope ratios, perhaps enhancing our ability to distinguish among areas within the Southern Lowlands.

The Guatemalan Highlands and Pacific Coast are a geologically young region, characterized by an extensive chain of volcanoes, some still active, that annually cover the area in ash, pumice, and other volcanic deposits. The Chortis block of the Highland area contains both Tertiary and Quaternary deposits [47, 48]. Strontium ratios from the area are influenced by a number of geologic factors, including recent volcanic activity and differential erosion that exposes older underlying bedrock in certain areas. Hodell and colleagues [17] identified ^87^Sr/^86^Sr values ranging from 0.70380 to 0.70492, which were the lowest values in the region of Mesoamerica examined in their study. Studies on ancient human skeletons in the area, such as at Kaminaljuyu (the ancient Maya center located beneath modern Guatemala City), reaffirm these low values [20]. Such strontium ratios are typical for subduction-related volcanism. Several geologic studies [49–54] have examined the strontium and lead content of igneous rocks in the volcanic region, identifying a distinctive range of lead ratios specific to particular regions of volcanic activity, a variation we expected to replicate with our analysis.

To the east of the Highlands lies the geologically diverse Metamorphic Province, containing a variety of rock types of different ages, including Paleozoic metamorphic rocks and Tertiary redbeds [55]. The Province has been further divided into several micro-regions based on its broad range of strontium isotope ratios (0.70417–0.72017), with some areas possessing higher values that reflect the Southern Lowlands, such as the Lake Izabal region, and others having lower ratios, closer to those of the Highlands, such as the Motagua River Valley. Price et al. [14] established a baseline for the Copan Valley using modern fauna, and found it to range from 0.70424 to 0.70735, on the lower end of the Metamorphic Province range. Lead ratios from the Metamorphic Province might be expected to exhibit similar variation, with the older Paleozoic rock having higher radiogenic lead content than the younger igneous rock that falls closer to the Volcanic Highlands in the west.

Finally, the Maya Mountains of southern Belize are characterized by Late Paleozoic rocks of various igneous, metamorphic, and sedimentary types [55]. These rocks exhibit some of the highest strontium ratios in Mesoamerica (0.71192–0.71514, as reported by Hodell et al. [17]), in part because of their older age. Thornton [11, 12] reported similar elevated strontium ratios in both ancient and modern fauna from the area, with some values reaching as high as 0.7316. In terms of our expectations for lead isotope ratios from sampled bedrock, these older Paleozoic rocks should have higher ratios than those in geologically younger regions.

## Materials and Methods

Sixty samples were chosen for lead isotope analysis. Of these, 36 were from non-weathered rock material collected by Hodell and colleagues for their study of strontium isotopes [17], 22 were collected by Gilli for strontium isotope testing from multiple sites inside and outside the Chicxulub crater basin [37], and two were collected by Sharpe for this study. Samples were collected from a variety of locations, including rock outcrops, road cuts, *cenotes*, and caves, and were chosen based on how representative they were of the local geology and on their proximity to known archaeological sites. GPS coordinates of the samples are provided in Table 1. Limestone ages were obtained using the strontium data with the method described by McArthur et al. [56]. In addition to these rock samples, nine modern plant samples and four soil samples were collected from the Yucatan by Gilli and used in this study to identify modern anthropogenic lead contamination from pollution. Samples collected for the Hodell et al. [17] and Gilli et al. [37] studies did not require permissions for geological sampling because the material collected did not include archaeological samples, and the plant samples collected did not include endangered or protected species. Samples were collected from Ceibal, Guatemala, under a permit to Sharpe from the Guatemala Institute of Anthropology and History (Instituto de Antropología e Historia de Guatemala).

**Table 1:** Lead and strontium results from the Mesoamerican samples used in this study.

Sample No.	Region	Sample Name/Location	Sample Type	^87^Sr/^86^Sr	^206^Pb/^204^Pb	^207^Pb/^204^Pb	^208^Pb/^204^Pb	Latitude	Longitude
1	Northern Lowlands	Dzilam de Bravo	limestone, Pleistocene	0.70907	19.097	15.661	38.769	21.37	-88.90
2	Northern Lowlands	Near Izamal	limestone, Pliocene	0.70906	19.144	15.685	38.800	20.94	-89.01
3	Northern Lowlands	Near Tecoh	limestone, Pliocene	0.70905	18.848	15.646	38.661	20.77	-89.47
4	Northern Lowlands	Holca	limestone, Miocene	0.70824	19.207	15.676	38.877	20.76	-88.93
5	Northern Lowlands	Yaxcopol	limestone, Pliocene	0.70897	19.319	15.697	38.977	20.75	-89.72
6	Northern Lowlands	Libre Union	limestone, Oligocene	0.70783	19.759	15.697	38.590	20.71	-88.80
7	Northern Lowlands	Yokdzonot	limestone, Miocene	0.70840	19.047	15.662	38.908	20.71	-88.73
8	Northern Lowlands	Piste	limestone, Miocene	0.70806	23.213	15.815	38.582	20.70	-88.60
9	Northern Lowlands	Kaua	limestone, Miocene	0.70847	19.105	15.671	38.904	20.62	-88.42
10	Northern Lowlands	Chemax	limestone	nd	19.073	15.669	38.810	20.66 (est)	-87.84 (est)
11	Northern Lowlands	Punta Laguna 1	limestone, Pleistocene	0.70908	18.855	15.649	38.815	20.65 (est)	-87.65 (est)
12	Northern Lowlands	Punta Laguna 2	limestone, Miocene	0.70812	18.957	15.667	38.848	20.65	-87.65
13	Northern Lowlands	Cuncunil	limestone, Miocene	0.70858	18.965	15.624	38.586	20.64	-88.30
14	Northern Lowlands	Entrance Mayapan	limestone, Pleistocene	0.70908	19.159	15.666	38.757	20.63	-89.46
15	Northern Lowlands	Cave Calcehtok	limestone, Miocene	0.70849	18.994	15.660	38.765	20.59	-89.90
16	Northern Lowlands	Cobá	limestone, Pliocene	0.70905	18.999	15.650	38.726	20.52	-87.65
17	Northern Lowlands	North of Muna	limestone	nd	19.278	15.679	38.821	20.51 (est)	-89.71 (est)
18	Northern Lowlands	South of Muna	limestone	nd	19.029	15.647	38.731	20.46 (est)	-89.71 (est)
19	Northern Lowlands	Tulum-Cobá	limestone	nd	18.926	15.667	38.874	20.36 (est)	-87.58 (est)
20	Northern Lowlands	Hobonil Rd 184	limestone	nd	19.113	15.662	38.836	20.45 (est)	-89.65 (est)
21	Northern Lowlands	Santa Elena-Ticul	limestone	nd	19.032	15.659	38.775	20.35 (est)	-89.60 (est)
22	Northern Lowlands	Cenote Cristal (near Tulum)	limestone, Pleistocene	0.70914	18.915	15.695	38.887	20.20	-87.50
23	Northern Lowlands	Cave Ixinche	limestone	nd	21.572	15.774	38.742	20.20 (est)	-87.50 (est)
24	Northern Lowlands	Sayil	limestone	nd	19.526	15.698	38.873	20.18 (est)	-89.65 (est)
25	Northern Lowlands	Xcambo	limestone, Pleistocene	0.70907	19.018	15.648	38.668	21.31	-89.36
26	Northern Lowlands	Cenote Azul Bacalar^a^	limestone, Miocene	0.70845	18.852	15.643	38.664	18.69	-88.38
27	Southern Lowlands	Palenque	limestone, Oligocene	0.70780	19.565	15.656	38.550	17.49	-92.05
28	Southern Lowlands	Uaxactun Grupo A	limestone, Oligocene	0.70778	25.519	15.813	38.547	17.40	-89.64
29	Southern Lowlands	El Pilar Comm. Creek trail	limestone, Upper Cretaceous	0.70766	19.857	15.662	38.507	17.23	-89.15
30	Southern Lowlands	Tikal in park	limestone, Oligocene	0.70779	21.961	15.768	38.565	17.22	-89.61
31	Southern Lowlands	Road to Tikal	limestone, Oligocene	0.70778	21.373	15.722	38.680	17.11	-89.68
32	Maya Mountains	Little Vaqueros River	granite	0.78347	19.743	15.726	39.389	17.04	-88.98
33	Southern Lowlands	Lake Sacpuy	limestone, Oligocene	0.70799	22.651	15.789	38.616	16.99	-90.05
34	Southern Lowlands	Lake Salpetén	limestone, Upper Cretaceous	0.70755	20.870	15.719	38.950	16.98	-89.68
35	Southern Lowlands	CA 13N. of Santa Ana Vieja	limestone, Upper Cretaceous	0.70737	21.955	15.763	38.644	16.67	-89.71
36	Southern Lowlands	Río San Juan	limestone, Oligocene	0.70777	20.667	15.691	38.730	16.63	-89.60
37	Southern Lowlands	Ceibal 1	limestone, Upper Cretaceous	0.70749	19.348	15.625	38.521	16.51	-90.06
38	Southern Lowlands	Ceibal 2	limestone, Upper Cretaceous	0.70747	19.510	15.617	38.496	16.51	-90.06
39	Southern Lowlands	Río Machaquila (Poptún)	limestone, Upper Cretaceous	0.70745	19.989	15.747	38.943	16.39	-89.44
40	Southern Lowlands	San Lucas	limestone, Oligocene	0.70777	30.966	16.189	38.768	16.36	-90.11
41	Southern Lowlands	Outcrop South of San Luis	claystone	0.70790	18.927	15.631	38.633	16.17	-89.41
42	Southern Lowlands	Candelaria	limestone, Upper Cretaceous	0.70733	19.159	15.659	38.659	15.88	-90.19
43	Southern Lowlands	San Simon II	limestone, Eocene	0.70773	45.623	16.944	38.855	15.83	-90.29
44	Motagua Valley (Metamorphic Province)	CA13-CA9 Intersection^b^	greenschist	0.70761	18.649	15.599	38.411	15.54	-88.84
45	Metamorphic Province	El Palacio	limestone, Oligocene	0.70789	18.963	15.658	38.697	15.36	-90.72
46	Metamorphic Province	Río Blanco^c^	slate	0.71690	18.879	15.675	39.233	15.33	-91.00
47	Metamorphic Province	Cunen III^c^	limestone, Miocene	0.70835	19.589	15.703	38.5975	15.32	-91.05
48	Metamorphic Province	Río Chixoy^c^	granite	0.71592	19.098	15.678	38.5285	15.27	-91.12
49	Motagua Valley (Metamorphic Province)	Río Huyus	serpentinite	0.70663	18.661	15.599	38.173	14.94	-89.83
50	Volcanic Highlands	Near Guaytan (ash)^d^	volcanic ash	0.70417	18.698	15.590	38.444	14.93	-89.97
51	Motagua Valley (Metamorphic Province)	Outcrop E of El Florida	felsic tuff	0.70580	18.868	15.649	38.734	14.86	-89.22
52	Motagua Valley (Metamorphic Province)	Chiquimula CA10	felsic tuff	0.70530	18.818	15.629	38.645	14.85	-89.52
53	Motagua Valley (Metamorphic Province)	Road to Copan	slate	0.70798	18.863	15.630	38.634	14.84	-89.19
54	Motagua Valley (Metamorphic Province)	CA 11	phyllite	0.74605	20.464	15.761	39.377	14.84	-89.35
55	Motagua Valley (Metamorphic Province)	Limestone quarry, km 62, CA9^c^	limestone	0.70528	18.286	15.590	37.823	14.83	-90.14
56	Volcanic Highlands	South of CA-1	andesite	0.70499	18.772	15.616	38.589	14.80	-91.11
57	Motagua Valley (Metamorphic Province)	Ticanlu Call	rhyolite	0.70689	18.790	15.644	38.695	14.74	-89.47
58	Volcanic Highlands	Lake Atitlán at Santiago III	basalt	0.70390	18.700	15.596	38.454	14.66	-91.20
59	Volcanic Highlands	Ciudad Vieja (outside Antigua)	volcanic ash	0.70392	18.688	15.595	38.432	14.53	-90.77
60	Volcanic Highlands	Río Esclavo^e^	basalt	0.70380	18.616	15.572	38.313	14.05	-90.34

^87^Sr/^86^Sr isotope ratios, with the exception of the samples from Ceibal, were previously reported in Gilli A, Hodell DA, Kamenov GD, Brenner M. Geological and archaeological implications of strontium isotope analysis of exposed bedrock in the Chicxulub crater basin, northwestern Yucatán, Mexico. Geol. 2009; 37: 723–726 and Hodell DA., Quinn RL, Brenner M, Kamenov G. Spatial variation of strontium isotopes (^87^Sr/^86^Sr) in the Maya region: A tool for tracking ancient human migration. J Archaeol Sci. 2004; 31: 585–601. Limestone ages obtained using the strontium data with the method described by McArthur JM, Howarth RJ, Bailey TR. LOWESS Version 3. Best-fit line to the marine Sr-isotope curve for 0 to 509 Ma and accompanying look-up table for deriving numerical age. Geol. 2001; 109: 155–169. The abbreviation *est*. denotes estimated latitude and longitude coordinates.

^a^Reported as Southern Lowlands in Hodell et al. (2004), but located near Northern Lowlands.

^b^Reported as Southern Lowlands in Hodell et al. (2004), but located in Metamorphic Province (Motagua Valley).

^c^Close to Volcanic Highlands.

^d^Reported as Metamorphic Province in Hodell et al. (2004), but located in Volcanic Highlands.

^e^Reported as Pacific Coast in Hodell et al. (2004), but located in Volcanic Highlands.

Rocks collected from the field were inspected and the exterior portion was removed. About 40–50 grams of non-weathered, fresh interior rock pieces were carefully selected for powdering. Carbonate samples were ground using ceramic mortars and pestles, thoroughly cleaned between samples. Silicate samples were ground using a Rocklabs bench-top ring mill (http://www.rocklabs.com). The first sample that was powdered was discarded and a fresh second sample was powdered and collected for analysis. Carbonate samples (30–50 mg) were dissolved in 2 ml of 50% nitric acid (HNO_3,_ Optima) in pre-cleaned Teflon vials, heated at 120°C on a hot plate for one hour, and then allowed to evaporate to dryness. Silicate samples (30–50 mg powder) were dissolved in 1 ml of Optima HNO_3_ and 2 ml hydrofluoric acid (HF, Optima) in Teflon vials, heated at 120°C on a hot plate for 24 hours, and then cooled before being allowed to evaporate to dryness.

In order to obtain modern bioavailable lead isotope values, soil samples were treated with 2 ml of weak 0.5N hydrochloric acid (HCl, Optima) in closed Teflon vials and heated at 80°C on a hot plate for 5 hours. The resultant leachate was removed and evaporated to dryness. In addition, five limestone leachates and residues were tested to compare whether the leached fraction was representative of the original limestone bedrock. The leached fraction was obtained by treating 100 mg of each bedrock sample with 2 ml 1N HCl (Optima) in closed pre-cleaned Teflon vials and heating the samples at 120°C on a hot plate for one hour. The residue was digested using the same method described above to dissolve the silicate rocks.

Lead from all samples was separated through ion chromatography using a Dowex 1X-8 resin with a 1N hydrobromic acid (HBr, Optima) wash and then collected with 20% HNO_3_ (Optima). Lead isotope ratios were obtained using a "Nu-Plasma" multiple-collector inductively-coupled-plasma mass spectrometer (MC-ICP-MS) in the Department of Geological Sciences at the University of Florida using Tl for mass-bias correction [57]. Values for the NBS 981 standard, analyzed with the samples and using the same Tl protocol in our lab, are as follows (n = 12): ^206^Pb/^204^Pb = 16.937 (±0.004, 2σ), ^207^Pb/^204^Pb = 15.490 (±0.003, 2σ), and ^208^Pb/^204^Pb = 36.695 (±0.009, 2σ). Individual sample errors are lower than the reproducibility of the NBS 981 standard. Samples were run, intermixed by rock type and region, on several separate occasions.

Lead concentration values were obtained for a representative subset of the samples. For this procedure, once the rock samples were dissolved and evaporated to dryness, 4 ml 5% HNO_3_ (Re-Rh) was added to each sample vial to dissolve the dry residue. A small amount of the solution was further diluted by weight with 5% HNO_3_ (Re-Rh) to a final dilution of 2000x and the samples were analyzed on an Element 2 ICP-MS. Quantification of the uranium, thorium, and lead concentrations was done by external calibration based on AGV-1, BCR-2, and BIR-1 USGS standards. The error of the reported uranium, thorium, and lead concentrations is less than 5%.

Cluster analysis was used to identify distinct regions of similar lead ratios. Cluster analysis is advantageous for identifying distinct homogeneous subgroups within a dataset. The Northern Lowlands, Southern Lowlands, and Motagua Valley samples were used for this analysis because they had the most data points. The analyses were performed using the K-means [58] algorithm in the statistical program R (version 3.1.1, [59]). The K-means algorithm attempts to fit all points to a number of clusters [58]. These clusters are determined by a number of operator-defined centroids, attempting to find the minimum number of clusters (*k*) that can accurately reflect the data. The algorithm places each centroid randomly in the dataset, assigns each data point to its closest centroid, then recalculates the position of the centroids until the distance between each point and one of the *k* centroids has been minimized using within-cluster sum of squared Euclidean distances, also referred to as the sum of squared error. The amount of within-cluster sum of squares variance decreases as more clusters are introduced, reaching 0 when the number of centroids is equal to the number of data points. The optimal number of clusters that represents the dataset lies at the juncture of where the within-cluster sum of squares variance begins to level out toward 0. Figures demonstrating how the number of K-means centroids was chosen using a scree plot for both ^207^Pb/^204^Pb and ^208^Pb/^204^Pb compared against ^206^Pb/^204^Pb for the Northern Lowlands, Southern Lowlands, and Motagua Valley are included in the S1 Appendix.

## Results

### Modern Yucatan Soil and Plant Samples

**Fig 2:**
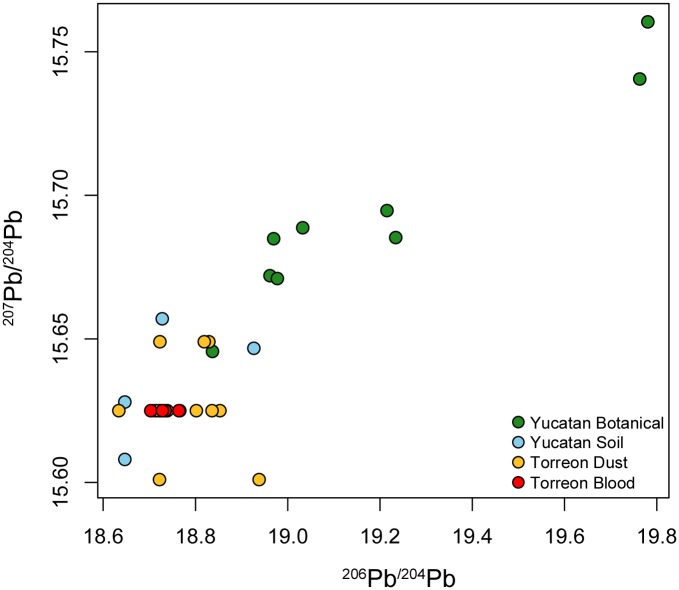
Modern Yucatan botanical and soil lead isotopic ratios compared with anthropogenic lead from the Torreón smelter. The Yucatan soil leachates show similar ratios to Torreón, Mexico, indicating they are likely contaminated by modern anthropogenic lead added to the local soils during the era of leaded gasoline usage. The plant samples form a trend from the soil leachates to higher ^207^Pb/^204^Pb and ^206^Pb/^204^Pb, indicating various degrees of anthropogenic lead contamination. The lead isotopic ratios for dust and blood samples from children in Torreón were previously reported by Soto-Jiménez MF, Flegal AR. Childhood lead poisoning from the smelter in Torreón, Mexico. Environ Res. 2011; 111: 590–596.

Table 2 shows the modern botanical and soil lead isotope ratios. The four soil leachates, representing bioavailable lead, overlap isotopically with anthropogenic lead isotope ratios reported from dust and human blood samples from children (Fig 2; Table 2) obtained from the urban area around the Met-Mex lead smelter facility in Torreón, Mexico [60]. The Torreón smelter is the largest lead production facility in Mexico, and was the principal source of lead for the leaded gasoline used in Mexico in the 20^th^ century [60]. As can be seen in Fig 2, the labile lead fractions in modern soils in the Yucatan area are isotopically similar to the Torreón smelter lead isotope ratios. The latter is representative of the lead used in leaded gasoline throughout Mexico. This indicates that bioavailable lead in the regional soils is dominated by anthropogenic lead released during the usage of leaded gasoline. The botanical samples from Mayapan, Uxmal, Xcambo, and Izamal form a trend extending from the modern soil leachates toward higher lead isotope ratios (Fig 2; Table 2). This trend suggests that the modern lead incorporated in plants in the region is representative of a mixture of natural bedrock lead and various amounts of anthropogenic lead that likely came from leaded gasoline. The banana leaves from Izamal, for example, exhibit the same lead ratios as those from Torreón, suggesting they are completely dominated by modern anthropogenic lead. The apparent randomness of the amount of modern anthropogenic lead incorporated in the plants is likely a consequence of factors such as root depth, access to contaminated soil horizons, and the phasing out of leaded gasoline in the near past. The lead isotope trend observed in the modern samples shows that we cannot use present-day soils and plants to establish reliable lead isotope baselines for archaeological investigations. The possibility may exist for using certain soil substrata from deeper contexts, particularly far from modern settlements or roads where leaded gasoline was used in the past, and this needs to be investigated further.

**Table 2:** Lead values from modern botanical and weak-acid soil leachate samples recovered from Yucatan, Mexico.

Sample	Location	^206^Pb/^204^Pb	^207^Pb/^204^Pb	^208^Pb/^204^Pb	Latitude	Longitude
Banana leaves	Izamal	18.837	15.646	38.407	20.93	-89.02
Banana, outer part	Mayapan Cave	19.215	15.695	38.717	20.63	-89.46
Banana, inner part	Mayapan Cave	19.780	15.760	39.058	20.63	-89.46
Grass	Xcambo	18.962	15.672	38.664	21.31	-89.36
Mangrove leaves	Xcambo	18.969	15.685	38.575	21.31	-89.36
Mangrove roots	Xcambo	18.977	15.671	38.588	21.31	-89.36
Maize leaves	Chacsinkin	19.033	15.689	38.593	20.17	-89.02
Maize leaves	Route to Uxmal	19.234	15.685	38.722	20.37	-89.77
Maize kernal	Route to Uxmal	19.763	15.741	39.037	20.37	-89.77
Soil 0.5N HCl Leachate	Izamal	18.647	15.608	38.472	20.93	-89.02
Soil 0.5N HCl Leachate	Mayapan	18.647	15.628	38.477	20.63	-89.46
Soil 0.5N HCl Leachate	Piste (Chichen Itza)	18.927	15.647	38.642	20.70	-88.60
Soil 0.5N HCl Leachate	Xcambo	18.728	15.657	38.599	21.31	-89.36

### Bedrock Samples from the Maya Region

**Fig 3:**
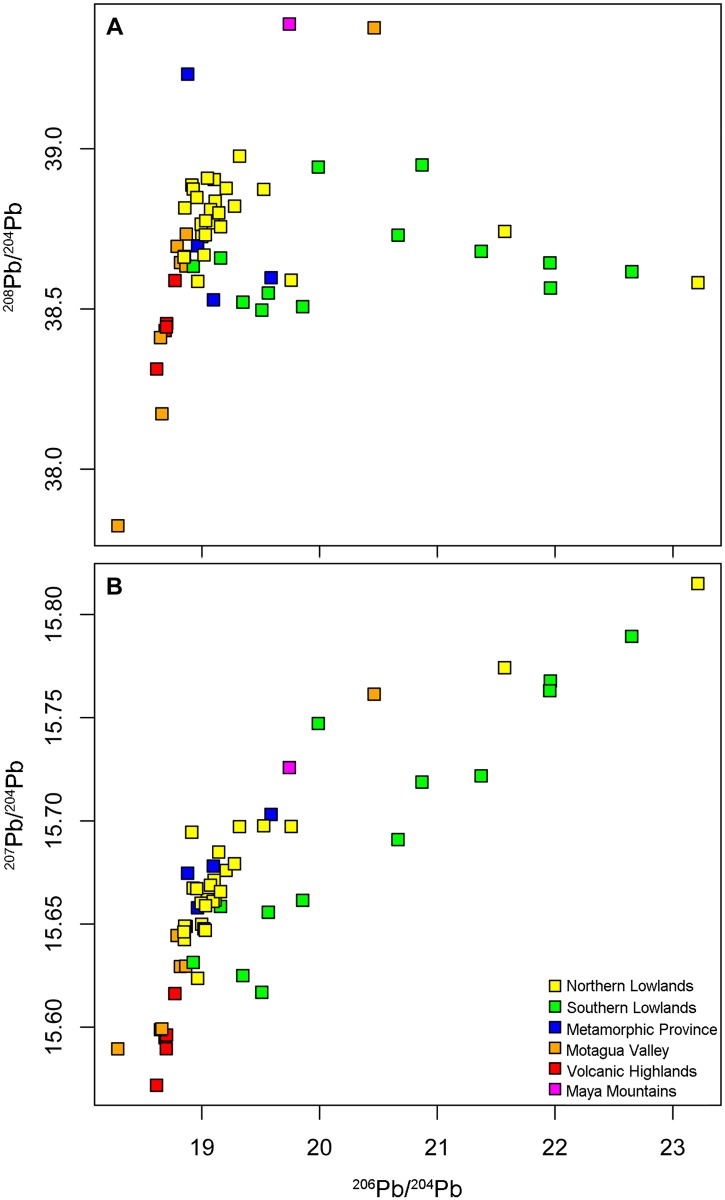
Comparative results for the lead isotope ratios. (A) ^208^Pb/^204^Pb and ^206^Pb/^204^Pb and (B) ^207^Pb/^204^Pb and ^206^Pb/^204^Pb. Outliers >24 for the ^206^Pb/^204^Pb are not shown as these highly radiogenic ratios are not likely to play a role in the overall bioavailable lead in the region because they exhibit low concentrations of lead (for more information, see text).

Table 1 and Fig 3 show the results for the lead isotope ratios obtained in this study to create the isotope ratio baselines. Among the three ratios of lead assessed from the rock samples, ^206^Pb/^204^Pb exhibits the widest range of variation, from 18.286 to 45.623, with lower values tending to concentrate in the Motagua Valley and Volcanic Highlands, and higher values characterizing limestone deposits in the Southern Lowlands. This considerable range indicates that there are substantial differences in radiogenic lead content throughout the Mesoamerican region and thus, the potential for greater specificity in sub-region distinction than provided by either oxygen or strontium isotopes alone.

Table 3 includes the five limestone leachates and residues that were tested to compare whether the HNO_3_-dissolved fraction was representative of the original limestone bedrock. The leaching experiments show that the residual silicate fractions are isotopically similar to the more soluble carbonate fractions. This shows that the leached portion of the bedrock would be the bioavailable component for living organisms, and would reflect the original bedrock’s isotope ratio values.

**Table 3:** Comparison of lead ratios obtained from limestone leachates with residues.

Sample	^206^Pb/^204^Pb	^207^Pb/^204^Pb	^208^Pb/^204^Pb	Latitude	Longitude
Hobonil Rd leachate	19.077	15.654	38.748	20.45 (est)	-89.65 (est)
Hobonil Rd residue	19.113	15.662	38.836	20.45 (est)	-89.65 (est)
Santa Elena-Ticul leachate	19.022	15.648	38.728	20.35 (est)	-89.60 (est)
Santa Elena-Ticul residue	19.020	15.659	38.775	20.35 (est)	-89.60 (est)
Cuncunil leachate	18.944	15.620	38.570	20.64	-88.30
Cuncunil residue	18.965	15.624	38.586	20.64	-88.30
Piste leachate	23.218	15.815	38.582	20.70	-88.60
Piste residue	23.213	15.808	38.585	20.70	-88.60
Near Izamal leachate	19.235	15.670	38.730	20.94	-89.01
Near Izamal residue	19.144	15.685	38.800	20.94	-89.01

The leachate represents the limestone fraction dissolved in 1N HCl. The residue was digested with concentrated HNO_3_ and HF. The abbreviation *est*. denotes estimated latitude and longitude coordinates.

The results of the concentration data (Table 4) reaffirm the pattern observed in the isotope ratios. The two Southern Lowland samples exhibiting elevated ^206^Pb/^204^Pb ratios, San Simon II and San Lucas, have two of the highest ^238^U/^204^Pb ratios, 1203 and 503, respectively. The Northern Lowlands also tended to exhibit high ^238^U/^204^Pb ratios, both areas being dominated by limestone. The lowest ratios were found in the Metamorphic Province, particularly the Motagua Valley. A serpentinite sample from Rio Huyus had a ^238^U/^204^Pb value of 1, the lowest in the sample set, which is reflected in its low lead isotope ratios as well.

**Table 4:** Lead, thorium, and uranium concentrations (ppm) in selected samples from the study area.

Sample No.	Region	Sample Name/Location	Sample Type	Pb	Th	U	^238^U/ ^204^Pb	Latitude	Longitude
3	Northern Lowlands	Near Tecoh	limestone, Pliocene	0.19	0.03	0.39	146	20.77	-89.47
6	Northern Lowlands	Libre Union	limestone, Oligocene	0.30	0.02	1.37	333	20.71	-88.80
7	Northern Lowlands	Yokdzonot	limestone, Miocene	1.04	0.53	0.36	25	20.71	-88.73
8	Northern Lowlands	Piste	limestone, Miocene	0.30	0.12	0.79	189	20.70	-88.60
11	Northern Lowlands	Punta Laguna 1	limestone, Pleistocene	2.08	1.25	0.87	30	20.65 (est)	-87.65 (est)
16	Northern Lowlands	Cobá	limestone, Pliocene	1.20	0.69	2.06	124	20.52	-87.65
19	Northern Lowlands	Tulum-Cobá	limestone	1.01	0.48	0.88	63	20.36 (est)	-87.58
20	Northern Lowlands	Hobonil Rd 184	limestone	2.43	1.36	0.25	8	20.45 (est)	-89.65 (est)
23	Northern Lowlands	Cave Ixinche	limestone	0.24	0.09	3.09	939	nd	nd
26	Northern Lowlands	Cenote Azul Bacalar	limestone, Miocene	0.69	0.26	0.50	52	18.69	-88.38
27	Southern Lowlands	Palenque	limestone, Oligocene	0.92	0.20	0.71	55	17.49	-92.05
28	Southern Lowlands	Uaxactun Grupo A	limestone, Oligocene	0.63	0.16	0.22	26	17.40	-89.64
30	Southern Lowlands	Tikal in park	limestone, Oligocene	0.41	0.06	0.25	44	17.22	-89.61
31	Southern Lowlands	Road to Tikal	limestone, Oligocene	0.58	0.15	0.08	10	17.11	-89.68
32	Maya Mountains	Little Vaqueros River	granite	19.5	31.9	13.7	51	17.04	-88.98
33	Southern Lowlands	Lake Sacpuy	limestone, Oligocene	0.63	0.16	1.69	193	16.99	-90.05
38	Southern Lowlands	Ceibal 2	limestone, Upper Cretaceous	2.27	1.30	1.06	34	16.51	-90.06
39	Southern Lowlands	Río Machaquila (Poptún)	limestone, Upper Cretaceous	4.02	0.59	2.57	46	16.39	-89.44
40	Southern Lowlands	San Lucas	limestone, Oligocene	0.80	0.07	5.58	503	16.36	-90.11
41	Southern Lowlands	Outcrop South of San Luis	claystone	3.74	1.60	0.95	18	16.17	-89.41
43	Southern Lowlands	San Simon II	limestone, Eocene	0.18	0.02	3.03	1203	15.83	-90.29
45	Metamorphic Province	El Palacio	limestone, Oligocene	3.15	0.19	0.81	19	15.36	-90.72
46	Metamorphic Province	Río Blanco	slate	10.7	13.2	3.29	22	15.33	-91.00
47	Metamorphic Province	Cunen III	limestone, Miocene	4.88	0.13	0.69	10	15.32	-91.05
48	Metamorphic Province	Río Chixoy	granite	22.8	5.04	1.39	4	15.27	-91.12
49	Motagua Valley (Metamorphic Province)	Río Huyus	serpentinite	0.97	0.02	0.02	1	14.94	-89.83
50	Volcanic Highlands	Near Guaytan (ash)	volcanic ash	12.7	11.1	4.44	25	14.93	-89.97
52	Motagua Valley (Metamorphic Province)	Chiquimula CA10	felsic tuff	11.0	11.1	4.46	29	14.85	-89.52
54	Motagua Valley (Metamorphic Province)	CA 11	phyllite	5.90	12.03	5.11	63	14.84	-89.35
55	Motagua Valley (Metamorphic Province)	Limestone quarry, km 62, CA9	limestone	0.54	0.15	0.16	21	14.83	-90.14
58	Volcanic Highlands	Lake Atitlán at Santiago III	basalt	7.46	3.73	1.42	14	14.66	-91.20
59	Volcanic Highlands	Ciudad Vieja (outside Antigua)	volcanic ash	13.8	8.20	2.40	13	14.53	-90.77

The abbreviation *est*. denotes estimated latitude and longitude coordinates.

**Fig 4:**
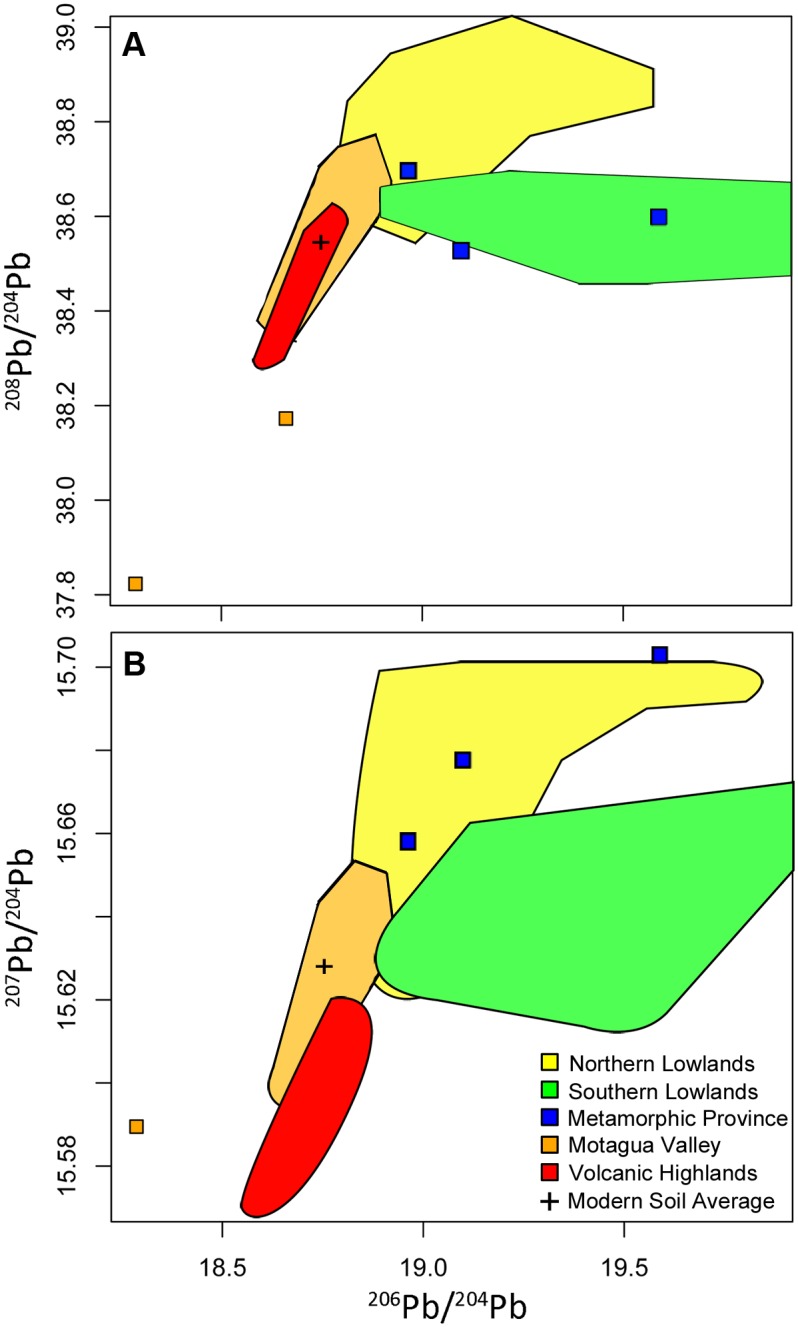
Ranges for the geographic regions, based on K-means cluster analyses. (A) ^208^Pb/^204^Pb and ^206^Pb/^204^Pb; (B) ^207^Pb/^204^Pb and ^206^Pb/^204^Pb. Modern anthropogenic lead is represented by the soil leachate isotopic average based on the four Yucatan samples reported in this study. Although included in the cluster analysis, the ^206^Pb/^204^Pb outliers above 20 are not shown in this graph in order to more clearly see the distinction between the other clusters.

**Fig 5:**
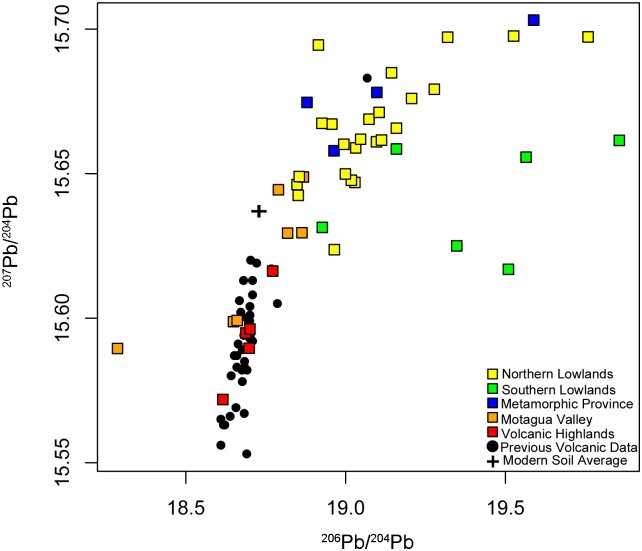
Results for ^207^Pb/^204^Pb and ^206^Pb/^204^Pb overlaid on previous data reported from the Guatemalan Volcanic Highlands. ^206^Pb/^204^Pb values >20 are not shown in order to more clearly discern the Volcanic Highlands data in relation to data from the other sub-regions. Previous data reported in Bardintzeff JM, Deniel C. Magmatic evolution of Pacaya and Cerro Chiquito volcanological complex, Guatemala. Bull Volcanol. 1992; 54: 267–283; Feigenson MD, Carr MJ, Maharaj SV, Juliano S, Bolge LL. Lead isotope composition of Central American volcanoes: Influence of the Galapagos plume. Geochem Geophys Geosys. 2004; 5(6): 1–14; Singer BS, Smith KE, Jicha BR, Beard BL, Johnson CM, Rogers NW. Tracking open-system differentiation during growth of Santa María Volcano, Guatemala. J Petrol. 2011; 52(12): 2335–2363; Walker JA, Carr MJ, Patino LC, Johnson CM, Feigenson MD, Ward RL. Abrupt change in magma generation processes across the Central American Arc in southeastern Guatemala: Flux-dominated melting near the base of the wedge to decompression melting near the top of the wedge. Contrib Mineral Petrol. 1995; 120(3): 378–390.

We grouped the lead isotope ratios from the rock samples using the geographic provinces that were used to assess the distribution of strontium isotopes in Mesoamerica by Hodell et al. [17] (Fig 4 and Table 5). The cluster analyses (S1 Appendix) support previous strontium ratio data in that rocks of similar ages tend to be located in the same area geographically. The Volcanic Highlands show the lowest overall ^207^Pb/^204^Pb compared to other provinces in the region. Our lead isotope data for the Volcanic Highlands are similar to published data for volcanic rocks from the region (Fig 5; [49, 52–54]). Two of the Motagua Valley samples overlap with the Volcanic Highlands, whereas, with the exception of one very low outlier, the rest plot at higher ^207^Pb/^204^Pb (Figs 4 and 5). The outlier, a limestone sample from a quarry, also has an unusually low strontium ratio (0.70528, as reported by Hodell et al. 2004 [17]). The Metamorphic Province samples show even higher ^207^Pb/^204^Pb and overlap with the Northern Lowlands. ^208^Pb/^204^Pb ratios in the Metamorphic Province are lower than in the Northern Lowlands and partially overlap with those from the Southern Lowlands. There is considerably more variation among the Southern Lowland samples across the ^206^Pb/^204^Pb range (18.927–45.623), with two samples, San Simon II (^206^Pb/^204^Pb = 45.623) and San Lucas (^206^Pb/^204^Pb = 30.966), serving as outliers on the higher end of the ^206^Pb/^204^Pb scale, as defined by cluster analysis (S1 Appendix). Overall, the Southern Lowland samples have significantly higher ^206^Pb/^204^Pb than even the maximum values for the other geographic regions. However, both the mean and the median of the Southern Lowlands plot at lower ^208^Pb/^204^Pb ratios when compared to the Northern Lowlands. The Motagua Valley has the broadest ^208^Pb/^204^Pb range (37.823–39.377). The single sample from the Maya Mountains can also be distinguished from the other geographic regions based on cluster analysis of all three lead isotope ratios and has the highest ^208^Pb/^204^Pb ratio of any sample (39.389), although it is not an extreme outlier as it was in the Hodell et al. [17] strontium study.

**Table 5:** Summary of statistics for the lead values.

		NL	SL	VH	MP	MP (MV)^a^	MM
Points		26	16	5	12	8	1
Minimum	206/204	18.848	18.927	18.616	18.286	18.286	--
	207/204	15.624	15.617	15.572	15.590	15.590	--
	208/204	38.582	38.496	38.313	37.823	37.823	--
Maximum	206/204	23.213	45.623	18.772	20.464	20.464	--
	207/204	15.815	16.944	15.616	15.761	15.761	--
	208/204	38.977	38.950	38.589	39.377	39.377	--
Mean	206/204	19.346	22.996	18.695	18.994	18.925	19.743
	207/204	15.676	15.812	15.594	15.651	15.638	15.726
	208/204	38.779	38.667	38.446	38.629	38.561	39.389
Median	206/204	19.060	20.768	18.698	18.865	18.804	19.743
	207/204	15.666	15.720	15.595	15.647	15.629	15.726
	208/204	38.788	38.638	38.444	38.639	38.639	39.389
Std. Dev. (1σ)	206/204	0.9485	6.7535	0.0553	0.5540	0.6502	--
	207/204	0.0400	0.3306	0.0159	0.0490	0.0547	--
	208/204	0.1061	0.1470	0.0979	0.4113	0.4542	--
Std. Error	206/204	0.1860	0.4221	0.0111	0.0462	0.0813	--
	207/204	0.0079	0.0207	0.0032	0.0041	0.0068	--
	208/204	0.0208	0.0092	0.0196	0.0343	0.0568	--

NL = Northern Lowlands, SL = Southern Lowlands, VH = Volcanic Highlands, MP = Metamorphic Province, MP (MV) = Motagua Valley, MM = Maya Mountains.

^a^Subset of the MP samples from the Motagua Valley and Copan.

A non-parametric Mann-Whitney U statistical test (Table 6) was used to compare the variability among ^206^Pb/^204^Pb, ^207^Pb/^204^Pb, and ^208^Pb/^204^Pb for all geographic regions except the Maya Mountains and Metamorphic Province samples outside of the Motagua Valley range, which had small sample sizes. The outlier limestone sample from the Motagua Valley rock quarry (Sample #55 on Table 1, “Limestone quarry, km 62, CA9”) that exhibited questionably low lead and strontium isotope ratios was also excluded. Mann-Whitney U was chosen as a means of analysis because it tests whether two independent sample datasets have the same distribution, and because it can be used with two small and unequally-sized samples [61, 62]. The null hypothesis tested is whether the two samples come from the same population, and therefore have the same or similar distributions. The Mann Whitney U test creates a U statistic for each dataset that is compared to the expected U statistic for the null hypothesis ([61] pages 52–54; see [63] for an extended table of values). Observations from both datasets are combined and ranked ordinally, starting at 1 for the lowest value. The equations for calculating the U statistic for the two sample datasets (x and y) are as follows:

$$U_{x}=n_{x}n_{y}+\left(\left(n_{x}\left(n_{x}+1\right)\right)/2\right)-R_{x}$$

$$U_{y}=n_{x}n_{y}+\left(\left(n_{y}\left(n_{y}+1\right)\right)/2\right)-R_{y}$$

**Table 6:** Results of the Mann-Whitney U Test.

	206/204		207/204		208/204	
	**NL**	**SL**	**NL**	**SL**	**NL**	**SL**
**N:**	26	16	26	16	26	16
**Mean Rank:**	9.607	11.893	11.845	9.655	15.762	5.738
**Mann-Whitney *U*:**	52.5 (expected = 143)		146.5 (expected = 143)		105 (expected = 143)	
***p*:**	5.95E-05*		0.1140		7.94E-03*	
	**NL**	**MV**	**NL**	**MV**	**NL**	**MV**
**N:**	26	7	26	7	26	7
**Mean Rank:**	15.242	1.758	15.136	1.864	14.758	2.242
**Mann-Whitney *U*:**	30 (expected = 53)		33.5 (expected = 53)		46 (expected = 53)	
***p*:**	7.72E-03*		0.0120*		0.0501	
	**NL**	**VH**	**NL**	**VH**	**NL**	**VH**
**N:**	26	5	26	5	26	5
**Mean Rank:**	15.516	0.484	15.516	0.484	15.452	0.548
**Mann-Whitney *U*:**	0 (expected = 33)		0 (expected = 33)		2 (expected = 33)	
***p*:**	5.34E-04*		5.30E-04*		7.89E-04*	
	**SL**	**MV**	**SL**	**MV**	**SL**	**MV**
**N:**	16	7	16	7	16	7
**Mean Rank:**	10.478	1.522	9.913	2.087	8.348	3.652
**Mann-Whitney *U*:**	7 (expected = 30)		20 (expected = 30)		56 (expected = 30)	
***p*:**	1.19E-03*		0.0177*		0.9734	
	**SL**	**VH**	**SL**	**VH**	**SL**	**VH**
**N:**	16	5	16	5	16	5
**Mean Rank:**	10.286	0.714	10.286	0.714	10.000	1.000
**Mann-Whitney *U*:**	0 (expected = 19)		0 (expected = 19)		6 (expected = 19)	
***p*:**	1.11E-03*		1.11E-03*		5.67E-03*	
	**MV**	**VH**	**MV**	**VH**	**MV**	**VH**
**N:**	7	5	7	5	7	5
**Mean Rank:**	4.583	1.917	5.0833	1.4167	4.500	2.000
**Mann-Whitney *U*:**	8 (expected = 6)		2 (expected = 6)		9 (expected = 6)	
***p*:**	0.1439		0.01468*		0.1939	

Expected U values from Milton 1963, where p ≤ 0.05. Asterisk (*) denotes significant p values. NL = Northern Lowlands, SL = Southern Lowlands, MP = Metamorphic Province (not including Motagua Valley), MV = Motagua Valley, VH = Volcanic Highlands. Motagua Valley sample #55 ("Limestone quarry, km 62, CA9") not included due to unreliable values. Maya Mountains not included due to single sample size.

Here, n_x_ is the number of observations in the first dataset, n_y_ is the number of observations in the second set, R_x_ is the sum of the ranks for dataset x’s observations, and R_y_ is the sum of the ranks for dataset y’s observations.

Variation within the Volcanic Highlands (n = 5) differs significantly (*p* < 0.05) when compared to both the Northern (n = 26) and Southern Lowlands (n = 16) for all three ratios. ^206^Pb/^204^Pb ratios exhibit the greatest variability among the regions tested, whereas ^208^Pb/^204^Pb ratios exhibited the most similarity, especially when comparing the Motagua Valley and other regions. The Northern Lowlands and Southern Lowlands differ significantly in both ^206^Pb/^204^Pb and ^208^Pb/^204^Pb. The Motagua Valley only differs significantly from the Volcanic Highlands for ^207^Pb/^204^Pb. Overall most regions do not exhibit significant overlap, with the exception of the Motagua Valley with the Volcanic Highlands and with ^208^Pb/^204^Pb for the other regions.

## Discussion

### Lead Isotope Distributions in the Maya Region

Although the introduction of anthropogenic lead changed the original lead isotope signal of modern soils as early as the Iron Age in Europe and particularly during the Industrial Revolution [42, 43, 45, 64], such contamination did not begin in Mesoamerica until the industrial age because of the limited amount of smelting activity that occurred in the area [33]. Previously, indigenous metalworking and other human inputs would have had little or no impact on the isotopic signature of naturally occurring lead. In the absence of anthropogenic input to archaeological materials in Mesoamerica, we can assume that the soil in a given region inherits its lead isotope signature from the underlying bedrock. Physical and chemical weathering are the dominant processes that incorporate lead from bedrock into the soil. Thus, artifacts containing soil and rock material, such as ceramics, should reflect these ratios; furthermore, humans and other organisms that inhale or ingest this soil, particularly as dust, will incorporate these lead ratios into their tissue (e.g., tooth enamel, bone). The lead isotope fields in Fig 4 should therefore represent the soil and bioavailable lead in each province during ancient times. Modern plants and soils may be contaminated by anthropogenic lead from pollution (Fig 2), and so cannot be used to establish reliable isotope ratio baselines for the period before industrial smelting activities began.

We find significant differences among some of the lead isotope ratios, particularly between the lowland and highland samples, a consequence of differences in the type and age of rocks between the two regions, but we also find some overlap as well, especially when samples from the Metamorphic Province and Motagua Valley are compared to other regions. This overlap is not unexpected, as the entire Metamorphic Province, including the Motagua Valley, is characterized by a variety of rock types with different ages, including young igneous rock in the transition zones with the Volcanic Highlands, and older metamorphic and sedimentary rock that dates to the Paleozoic Era. Distinctions among the geographic regions are clearest when all three lead isotope ratios are compared simultaneously, emphasizing the value of the three lead isotope ratios for future archaeological applications, in combination with other lines of evidence.

Of all the regions, the Southern Lowlands exhibit the greatest range of variation of ^206^Pb/^204^Pb and ^207^Pb/^204^Pb, and may have the best potential for identifying patterns of migration and exchange in the archaeological record within the province. Both ^206^Pb/^204^Pb and ^207^Pb/^204^Pb are, on average, higher in this region than in the other geographic areas (Fig 3 and Table 5). The two ^206^Pb/^204^Pb lowland outliers, San Simon II and San Lucas, are not located near one another and suggest the existence of distinct, highly radiogenic domains throughout this region that can be useful for more detailed geo-referencing. Northeastern Guatemala, particularly around the archaeological site of Uaxactun and the Lake Petén Itzá region, also has elevated (>20) and particularly variable ^206^Pb/^204^Pb ratios. Such variation could prove useful in archaeological studies because this area was once densely populated and had been an important trade hub during the ancient Maya Preclassic and Classic periods (*ca*. 2000 BCE—CE 1000). Overall, all but one sample from the Southern Lowlands show ^206^Pb/^204^Pb>19 (Table 1). The single sample that exhibits the low ^206^Pb/^204^Pb value was the only claystone sample used in the study, which may explain its unique signature, whereas all other Southern Lowlands samples were carbonate rocks. Given the distinctive nature of this sample, it is unlikely to significantly influence the lead isotopic composition of the soils developed over the carbonate bedrock.

Elevated ^206^Pb/^204^Pb and ^207^Pb/^204^Pb ratios in the Southern Lowlands are a result of high, time-integrated U/Pb ratios. Some of the carbonate rocks analyzed in this work show ^238^U/^204^Pb above 100, with San Simon II showing extremely high ^238^U/^204^Pb (1203, Table 4). Therefore, as a result of U-decay, the ^206^Pb/^204^Pb ratios, and to some extent the ^207^Pb/^204^Pb ratios, increased rapidly since the initial deposition of the Late Cretaceous-Paleocene limestones, particularly in the case of San Simon II. In general, carbonate rocks do not show high Th/Pb ratios (for example, San Simon II has Th/Pb = 0.11 compared to U/Pb = 16.83), and this is why there is no concomitant increase in ^208^Pb/^204^Pb.

There is less variation among the samples from the Northern Lowlands, despite the fact that the geology is also dominated by carbonate rocks and some of them show elevated U/Pb (Table 4). This is a consequence of the younger depositional age (less time for uranium-decay to affect lead isotopes) compared to the Southern Lowlands. Although lead isotope ratios from the Northern Lowlands partially overlap with those from the other geographic regions, when all three isotope ratios are compared, they tend to fall within a distinct cluster (Figs 3 and 4). There are only two outliers, Piste and Cave Ixiche, which plot at high ^206^Pb/^204^Pb. Similar to some of the carbonate samples from the Southern Highlands, these two samples have very high U/Pb ratios. Piste shows ^238^U/^204^Pb = 189 and Cave Ixinche shows ^238^U/^204^Pb = 939 (Table 4). Although Cave Ixinche shows higher U/Pb, the ^206^Pb/^204^Pb is lower when compared to Piste (Table 1). This indicates episodes of disturbance of the U/Pb system since deposition. It is likely that multiple geological events, such as diagenesis, uplift, and karst development have disturbed the U/Pb systematics of the carbonates. Although such events can affect the lead isotope compositions of limestone on geological time-scales, we can assume that the present-day lead isotope composition is the same as it was when the first humans settled the region several thousand years ago. Furthermore, such samples with very high ^206^Pb/^204^Pb are overall on the low end in terms of lead concentrations (Table 4) and therefore are not a significant component of the average lead isotopic composition of the Northern Lowlands region. When compared to the Southern Lowlands, the Northern Lowlands form a cluster characterized by lower ^206^Pb/^204^Pb, higher ^207^Pb/^204^Pb when ^206^Pb/^204^Pb is less than 20, and overall higher ^208^Pb/^204^Pb (Fig 4). The Northern Lowlands overlap slightly on the low-end with the most radiogenic lead ratios of the Motagua Valley, although the two regions are geographically distinct. There is no overlap between the Northern Lowlands and the Volcanic Highlands in terms of ^206^Pb/^204^Pb and ^207^Pb/^204^Pb, and barely any for ^208^Pb/^204^Pb (a difference of 0.007).

The Motagua Valley exhibits the greatest amount of variation for ^208^Pb/^204^Pb, but less so for ^206^Pb/^204^Pb and ^207^Pb/^204^Pb (Fig 3 and Table 5). Cluster analysis shows that the less radiogenic Motagua Valley samples overlap as a cluster with the Volcanic Highlands, and it may prove difficult to distinguish between these two regions with respect to potential movement of artifacts and/or people in the past. This overlap is in part a consequence of the presence of volcanic material in the Motagua region. Although the Metamorphic Province is a region of mixed rock types including metamorphic material dating to the Paleozoic Era, due to its border with the Volcanic Highlands part of the region contains much younger volcanic rocks like the felsic tuff and rhyolite used in this study. Such diverse isotopic variation was reported previously using strontium isotope analysis on local wildlife in the valley ([14] page 27).

There are two notable outliers among the Motagua samples (Fig 3). The high ^208^Pb/^204^Pb value is a specimen of phyllite, a metamorphic rock, and the only rock of this type used in the study. Most likely this sample has affinity to the Metamorphic Province. The other Motagua outlier, a limestone sample collected from a quarry, is the lowest ^206^Pb/^204^Pb sample analyzed in this study (Figs 3 and 4). The lead and strontium signals in this sample are highly anomalous, given the elevated ^206^Pb/^204^Pb and ^87^Sr/^86^Sr observed in the carbonate rocks in the region. Furthermore, its strontium ratio (0.70528) is virtually impossible for Phanerozoic limestone and therefore we did not consider the lead isotopes of this sample in the data analyses.

Only five Volcanic Highland samples were tested in this study, including two samples of volcanic ash. All five overlap with lead ratios obtained from previous studies of volcanic rocks in the region (Fig 5). With the exception of two Motagua Valley samples, the Volcanic Highland samples from this study do not overlap with sample values from the other geographic areas, making them very useful for distinguishing between the highlands, terrain characterized by volcanic rocks, and the lowlands, characterized by much older carbonate rocks. It is noteworthy that the volcanic lead isotope ratios overlap significantly with the values obtained from the modern contaminated botanical and soil samples with respect to ^208^Pb/^204^Pb, but not ^207^Pb/^204^Pb. This partial overlap should not be problematic when sourcing archaeological material, mainly human and animal tooth enamel, which is the most commonly used sourcing material for lead and strontium isotope studies. This is because these materials are fairly resistant to post-mortem contamination [23, 44], such as the widespread pollution from modern anthropogenic lead that occurred during the 20th century.

The Maya Mountains are characterized by late Paleozoic and Mesozoic rocks that range in age from 320 to 125 million years ([17] page 594), and would therefore be expected to have higher lead ratios than the younger igneous rocks derived from the Volcanic Highlands and Motagua Valley. Only one sample from the Maya Mountains, however, was assayed because only one bedrock sample was collected for the original Hodell et al. [17] strontium study. The other samples in that study were water. This sample exhibited higher ^208^Pb/^204^Pb ratios than most of the other samples in the study (Fig 3), with the exception of one sample from the Metamorphic Province and the phyllite outlier from the Motagua Valley. The ^208^Pb/^204^Pb for the Maya Mountains sample is also elevated in comparison to the ^208^Pb/^204^Pb for the Southern Lowlands. Additional sampling and more data would be needed to substantiate the variability of lead ratios in the Maya Mountains region.

### Comparison of Lead and Strontium Isotope Distributions

**Fig 6:**
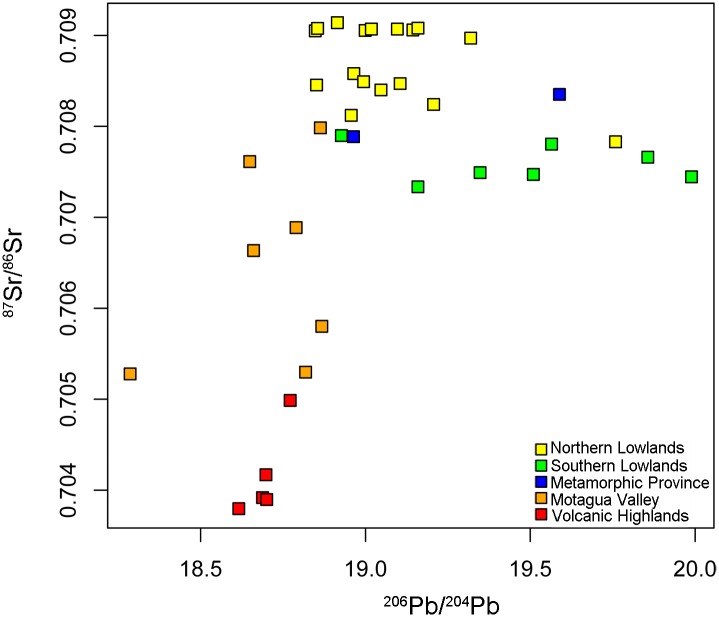
Comparison of ^87^Sr/^86^Sr with ^206^Pb/^204^Pb. ^206^Pb/^204^Pb values >20 are not shown. Strontium values were previously reported in Hodell DA., Quinn RL, Brenner M, Kamenov G. Spatial variation of strontium isotopes (^87^Sr/^86^Sr) in the Maya region: A tool for tracking ancient human migration. J Archaeol Sci. 2004; 31: 585–601 and Gilli A, Hodell DA, Kamenov GD, Brenner M. Geological and archaeological implications of strontium isotope analysis of exposed bedrock in the Chicxulub crater basin, northwestern Yucatán, Mexico. Geol. 2009; 37: 723–726.

The lead isotope results complement the previous measurements of strontium isotopes by Hodell et al. [17], and provide an additional proxy with increased detail for assessing trade and human mobility in the past (Fig 6 and Table 1). The most significant difference between the strontium and lead results is the considerable variation in the Southern Lowlands lead ratios, particularly for ^206^Pb/^204^Pb. The latter is a consequence of high and variable time-integrated U/Pb in the limestones, as discussed above. In contrast, this region exhibits far less variation in ^87^Sr/^86^Sr, because of high strontium, but very low rubidium in the carbonates. In terms of strontium isotope ratios, the Southern Lowlands are intermediate between strontium isotope ratios in the Northern Lowlands and the Volcanic Highlands/Motagua Valley regions, although they are typically much higher than these regions in terms of lead, especially ^206^Pb/^204^Pb. Thus, lead ratios provide a novel means to distinguish the Southern Lowlands from other regions, and also potentially permit discrimination among different sub-regions within the Southern Lowlands.

Lead isotopes are particularly useful because the Southern Lowlands were the heartland of the Preclassic and Classic Maya culture, where previous efforts to discern micro-regions using oxygen and strontium were often unsuccessful because of the homogeneity of isotopic ratios across the region. For example, lead and strontium ratios from the Petexbatun area of Guatemala tend to be lower than those of the Lake Petén Itzá region in the northeast of the country. It is known from hieroglyphic inscriptions on monuments and ceramic artifacts that the royal elites visited and moved between these two regions (e.g. [65] page 227; [66] page 327), and the combined use of lead and strontium may provide a means to identify the objects and people who moved between these areas. Another possible example of “in-province” differences are the Salpeten and Machaquila samples in the Southern Lowlands (Table 1). These two samples show distinctly higher ^208^Pb/^204^Pb values compared to the rest of the Southern Lowlands (Fig 3 and Table 1). Although these two samples, located in northeast Guatemala, overlap in lead isotope space with the Northern Lowlands, they have the low ^87^Sr/^86^Sr values characteristic of the Southern Lowlands. Therefore, there is a potential to distinguish within-province migration when strontium and lead isotopes are used in combination.

Combined use of lead and strontium ratios can also differentiate among the Metamorphic Province/Motagua Valley, the Southern Lowlands, and the Northern Lowlands. On occasion the Metamorphic Province and Southern Lowlands strontium ratio baselines obtained in previous studies overlap [11, 12, 17]. This has also been noted in areas such as the Copan Valley within the Motagua region, where strontium isotope baseline values exhibit a broad range of ratios [14]. A combination of strontium and lead ratios from all three areas, however, reveals more clearly distinct differences among the three regions (Fig 6), and thus allows us to source bone and artifactual material with a much greater level of confidence than was previously possible.

Although strontium ratios provide an excellent means to distinguish the Maya Mountains from the rest of the Mesoamerican area, this study demonstrates that lead ratios from this region can be conflated with those from the Southern Lowlands, particularly in terms of ^206^Pb/^204^Pb and ^207^Pb/^204^Pb isotopes. Thus, although lead ratios may distinguish the Maya Mountains from most other geographic areas because of the distinct geology of the mountains, strontium ratios may be better to differentiate between the mountains and the nearby Southern Lowlands, at least until more data are obtained. Overall, the combination of strontium and all three lead isotope ratios is the most powerful means to track the movement of humans, animals, and objects in the Maya region.

## Conclusions

Because of the diverse rock types and ages that span the Maya region, lead isotope ratios hold the potential to serve as a powerful tool for sourcing human, animal and other archaeological remains. Within the region settled by the ancient Maya, five distinct provinces, including the Northern and Southern Lowlands, the Motagua Valley, the Metamorphic Province, and the Volcanic Highlands, can be distinguished based on a combination of lead and strontium isotope analyses of bedrock samples. The Northern and Southern Lowlands show overall higher ^206^Pb/^204^Pb ratios compared to the other three provinces. Although there is overlap between the two areas in ^206^Pb/^204^Pb, the Northern Lowlands shows higher ^208^Pb/^204^Pb compared to the Southern Lowlands. The Volcanic Highlands, overall, exhibit the lowest ^207^Pb/^204^Pb ratios in the region. The Motagua Valley lead isotopes overlap to some extent with the Volcanic Highlands because volcanic material naturally transitions into a largely metamorphic base in the rocks that characterize the river valley. The Metamorphic Province shows overlapping ^206^Pb/^204^Pb ratios with the two lowland areas. It has, however, ^208^Pb/^204^Pb ratios distinct from the Northern Lowlands and ^207^Pb/^204^Pb ratios distinct from the Southern Lowlands.

When used with other sourcing techniques, such as strontium and oxygen isotopes, lead isotope values have the potential to distinguish among geographic regions at a finer spatial scale than was previously possible. This study only assessed bedrock samples from the Yucatan, Guatemala, Belize, and Honduras regions, and further analysis outside this geographic area is necessary to identify broader trade and migration networks, which extended into central Mexico and southward into El Salvador. Further lead isotopic testing across the landscape may also clarify certain geologically distinct regions, such as the Maya Mountains in Belize. Future development of a lead isoscape and the application of lead isotope measures on archaeological materials will enable better inferences about the mobility of humans and the movement and trade of archaeological materials across Mesoamerica, and enhance our understanding of the dynamic Maya civilization.

## Supporting Information

S1 Appendix K-means cluster analysis reports for the Northern Lowlands, Southern Lowlands, and Motagua Valley. (A) K-Means cluster analysis for the Northern Lowlands, ^207^Pb/^204^Pb and ^206^Pb/^204^Pb. Black and red colors designate different clusters, identified by centroid numbers. (B) K-Means within-cluster sum of squares scree plot for the Northern Lowlands, ^207^Pb/^204^Pb and ^206^Pb/^204^Pb. The “elbow”, defined by the arrow, denotes the optimal number of clusters, in this case 2 (*k* = 2). (C) K-Means cluster analysis for the Northern Lowlands, ^208^Pb/^204^Pb and ^206^Pb/^204^Pb. Black and red colors designate different clusters, identified by centroid numbers. (D) K-Means within-cluster sum of squares scree plot for the Northern Lowlands, ^208^Pb/^204^Pb and ^206^Pb/^204^Pb. The “elbow”, defined by the arrow, denotes the optimal number of clusters, in this case 2 (*k* = 2). (E) K-Means cluster analysis for the Southern Lowlands, ^207^Pb/^204^Pb and ^206^Pb/^204^Pb. Black, red, and green colors designate different clusters, identified by centroid numbers. (F) K-Means within-cluster sum of squares scree plot for the Southern Lowlands, ^207^Pb/^204^Pb and ^206^Pb/^204^Pb. The “elbow”, defined by the arrow, denotes the optimal number of clusters, in this case a value between 2 and 3. The number of clusters is rounded up in this case (*k* = 3). (G) K-Means cluster analysis for the Southern Lowlands, ^208^Pb/^204^Pb and ^206^Pb/^204^Pb. Black, red, and green colors designate different clusters, identified by centroid numbers. (H) K-Means within-cluster sum of squares scree plot for the Southern Lowlands, ^208^Pb/^204^Pb and ^206^Pb/^204^Pb. The “elbow”, defined by the arrow, denotes the optimal number of clusters, in this case a value between 2 and 3. The number of clusters is rounded up in this case (*k* = 3). (I) K-Means cluster analysis for the Motagua Valley, ^207^Pb/^204^Pb and ^206^Pb/^204^Pb. Black and red colors designate different clusters, identified by centroid numbers. (J) K-Means within-cluster sum of squares scree plot for the Motagua Valley, ^207^Pb/^204^Pb and ^206^Pb/^204^Pb. The “elbow”, defined by the arrow, denotes the optimal number of clusters, in this case 2 (*k* = 2). (K) K-Means cluster analysis for the Motagua Valley, ^208^Pb/^204^Pb and ^206^Pb/^204^Pb. Black, red, and green colors designate different clusters, identified by centroid numbers. (L) K-Means within-cluster sum of squares scree plot for the Motagua Valley, ^208^Pb/^204^Pb and ^206^Pb/^204^Pb. The “elbow”, defined by the arrow, denotes the optimal number of clusters, in this case 3 (*k* = 3).
(PDF)
