# Supplementary material for: Lead (Pb) Isotope Baselines for Studies of Ancient Human Migration and Trade in the Maya Region
Source: PLoS One. 2016 Nov 2;11(11):e0164871. doi: 10.1371/journal.pone.0164871 (PMC5091867; doi:10.1371/journal.pone.0164871)
Supplement: S1 Appendix — (A) K-Means cluster analysis for the Northern Lowlands, 207Pb/204Pb and 206Pb/204Pb. Black and red colors designate different clusters, identified by centroid numbers. (B) K-Means within-cluster sum of squares scree plot for the Northern Lowlands, 207Pb/204Pb and 206Pb/204Pb. The “elbow”, defined by the arrow, denotes the optimal number of clusters, in this case 2 (k = 2). (C) K-Means cluster analysis for the Northern Lowlands, 208Pb/204Pb and 206Pb/204Pb. Black and red colors designate different clusters, identified by centroid numbers. (D) K-Means within-cluster sum of squares scree plot for the Northern Lowlands, 208Pb/204Pb and 206Pb/204Pb. The “elbow”, defined by the arrow, denotes the optimal number of clusters, in this case 2 (k = 2). (E) K-Means cluster analysis for the Southern Lowlands, 207Pb/204Pb and 206Pb/204Pb. Black, red, and green colors designate different clusters, identified by centroid numbers. (F) K-Means within-cluster sum of squares scree plot for the Southern Lowlands, 207Pb/204Pb and 206Pb/204Pb. The “elbow”, defined by the arrow, denotes the optimal number of clusters, in this case a value between 2 and 3. The number of clusters is rounded up in this case (k = 3). (G) K-Means cluster analysis for the Southern Lowlands, 208Pb/204Pb and 206Pb/204Pb. Black, red, and green colors designate different clusters, identified by centroid numbers. (H) K-Means within-cluster sum of squares scree plot for the Southern Lowlands, 208Pb/204Pb and 206Pb/204Pb. The “elbow”, defined by the arrow, denotes the optimal number of clusters, in this case a value between 2 and 3. The number of clusters is rounded up in this case (k = 3). (I) K-Means cluster analysis for the Motagua Valley, 207Pb/204Pb and 206Pb/204Pb. Black and red colors designate different clusters, identified by centroid numbers. (J) K-Means within-cluster sum of squares scree plot for the Motagua Valley, 207Pb/204Pb and 206Pb/204Pb. The “elbow”, defined by the arrow, denotes the optimal number o [file pone.0164871.s001.pdf]

Supporting Information

Generated by R (version 3.1.1)
Metamorphic Province (non-Motagua Valley) and Volcanic Highlands excluded due to each having only 4 and 5 data points, respectively.

Key:

centroid = average of all points in a group
SS = Sum of Squares (between_SS/total_SS = 100% is perfect fit)

Northern Lowlands:

$^{207}Pb/^{206}Pb$:

K-Means Report:
Centroids = 2
Cluster means:

	$^{206}Pb/^{204}Pb$	$^{207}Pb/^{204}Pb$
1	22.39229	15.79460
2	19.09218	15.66569

Within cluster sum of squares by cluster:
1.346978, 1.049611
(between_SS/total_SS = 89.4%)

$^{208}Pb/^{206}Pb$:

K-Means Report:
Centroids = 2
Cluster means:

	$^{206}Pb/^{204}Pb$	$^{208}Pb/^{204}Pb$
1	22.39229	38.66207
2	19.09218	38.78843

Within cluster sum of squares by cluster:
1.358936, 1.280167
(between_SS/total_SS = 88.4 %)

Southern Lowlands:

$^{207}Pb/^{206}Pb$:

K-Means Report:
Centroids = 3
Cluster means:
$^{206}Pb/^{204}Pb$ $^{207}Pb/^{204}Pb$

	$^{206}Pb/^{204}Pb$	$^{207}Pb/^{204}Pb$
1	45.62330	16.94360
2	28.24225	16.00115
3	20.44843	15.69600

Within cluster sum of squares by cluster:
0.00000, 14.90283, 17.94353
(between_SS /total_SS = 95.2%)

$^{208}Pb/^{206}Pb$:

K-Means Report:
Centroids = 3
Cluster means:

	$^{206}Pb/^{204}Pb$	$^{208}Pb/^{204}Pb$
1	20.44843	38.65338
2	28.24225	38.65780
3	45.62330	38.85490

Within cluster sum of squares by cluster:
18.16371, 14.85656, 0.00000
(between_SS /total_SS = 95.2%)

Motagua Valley:

$^{207}Pb/^{206}Pb$:

K-Means Report:
Centroids = 2
Cluster means:

	$^{206}Pb/^{204}Pb$	$^{207}Pb/^{204}Pb$
1	20.46376	15.76137
2	18.70488	15.61996

Within cluster sum of squares by cluster:
0.0000000, 0.2554385
(between_SS/total_SS = 91.4 %)

$^{208}Pb/^{206}Pb$:

K-Means Report:
Centroids = 3
Cluster means:

	$^{206}Pb/^{204}Pb$	$^{208}Pb/^{204}Pb$
1	18.47330	37.99805
2	18.79751	38.62367
3	20.46376	39.37748

Within cluster sum of squares by cluster:
0.13116280, 0.09497238, 0.00000000
(between_SS/total_SS = 94.9 %)

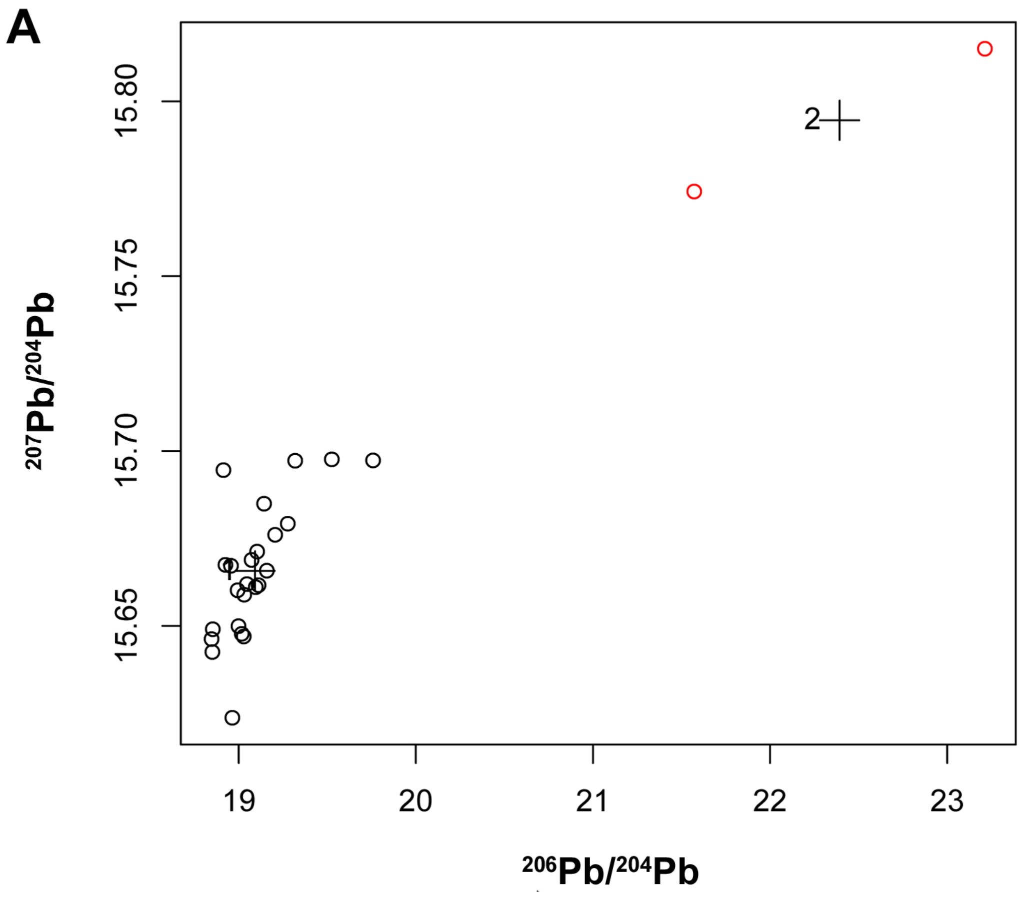
A
15.80
15.75
15.70
15.65
207Pb/204Pb
2
1
19
20
21
22
23
206Pb/204Pb

B

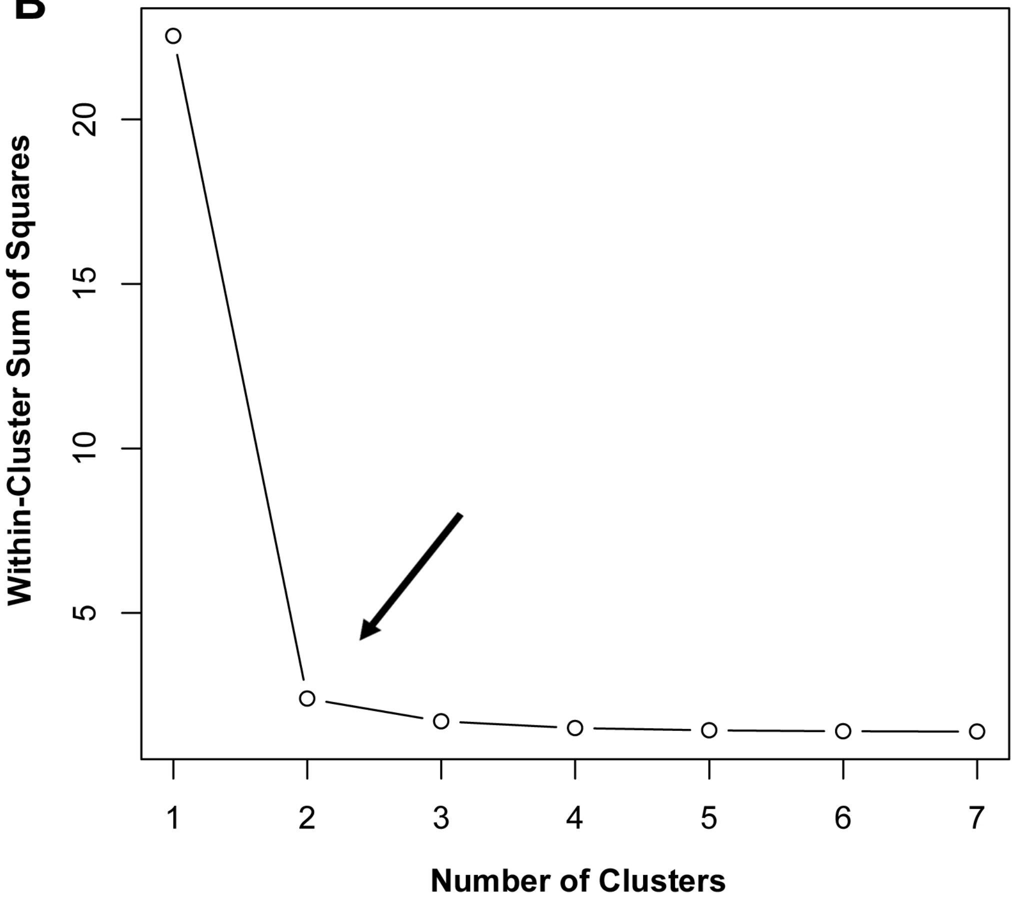
Within-Cluster Sum of Squares
20
15
10
5
1
2
3
4
5
6
7
Number of Clusters

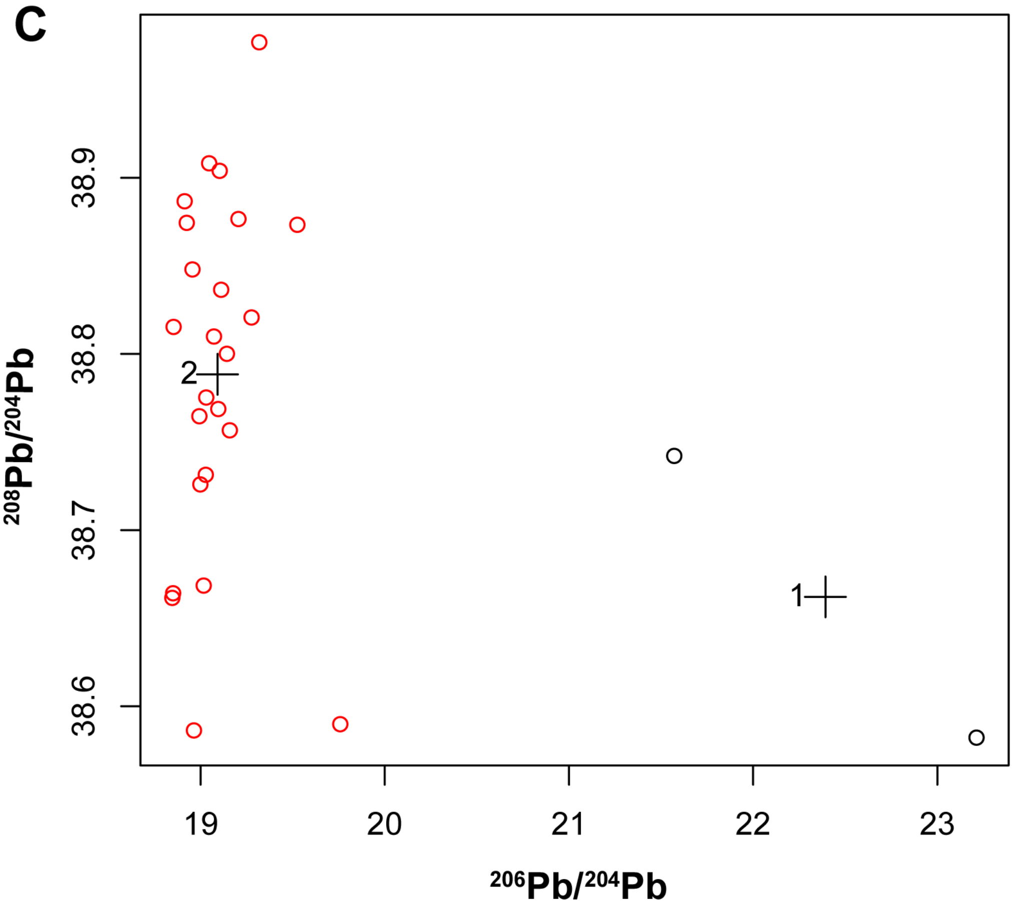
C
38.9
38.8
38.7
38.6
2
1
19
20
21
22
23
$^{206}Pb/^{204}Pb$
$^{208}Pb/^{204}Pb$

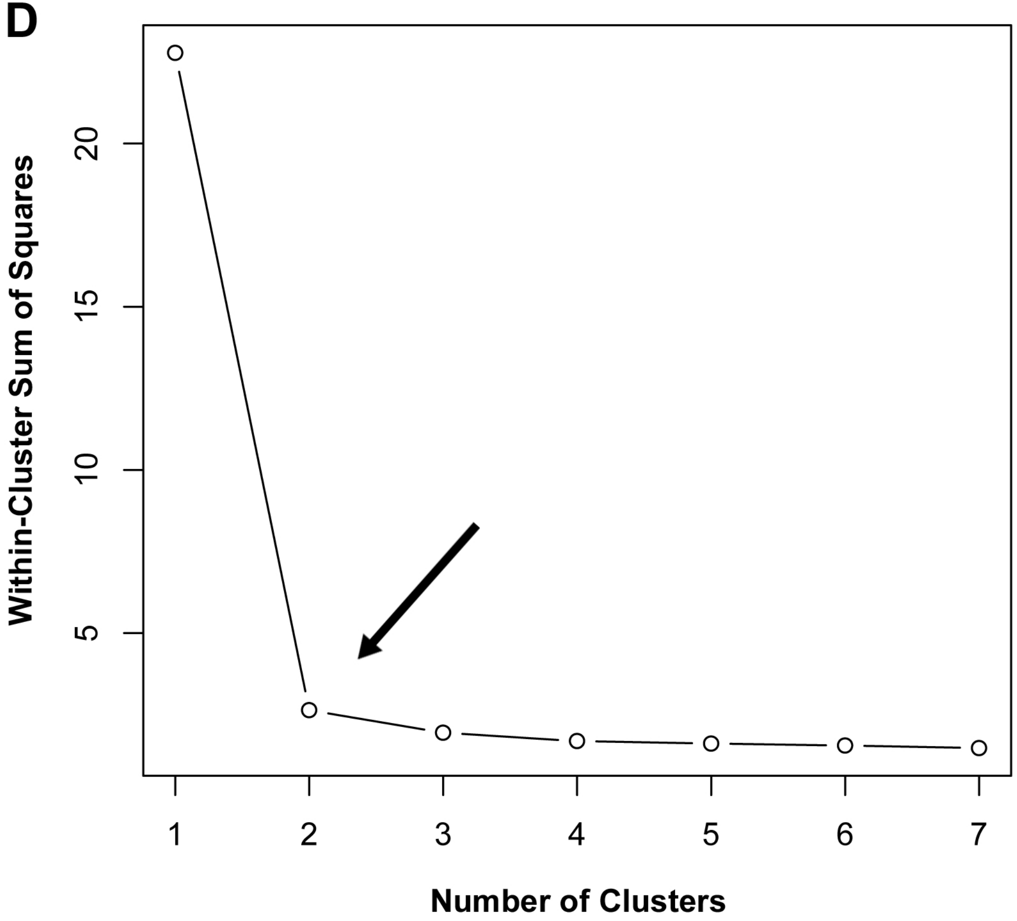
D
Within-Cluster Sum of Squares
5
10
15
20
1
2
3
4
5
6
7
Number of Clusters

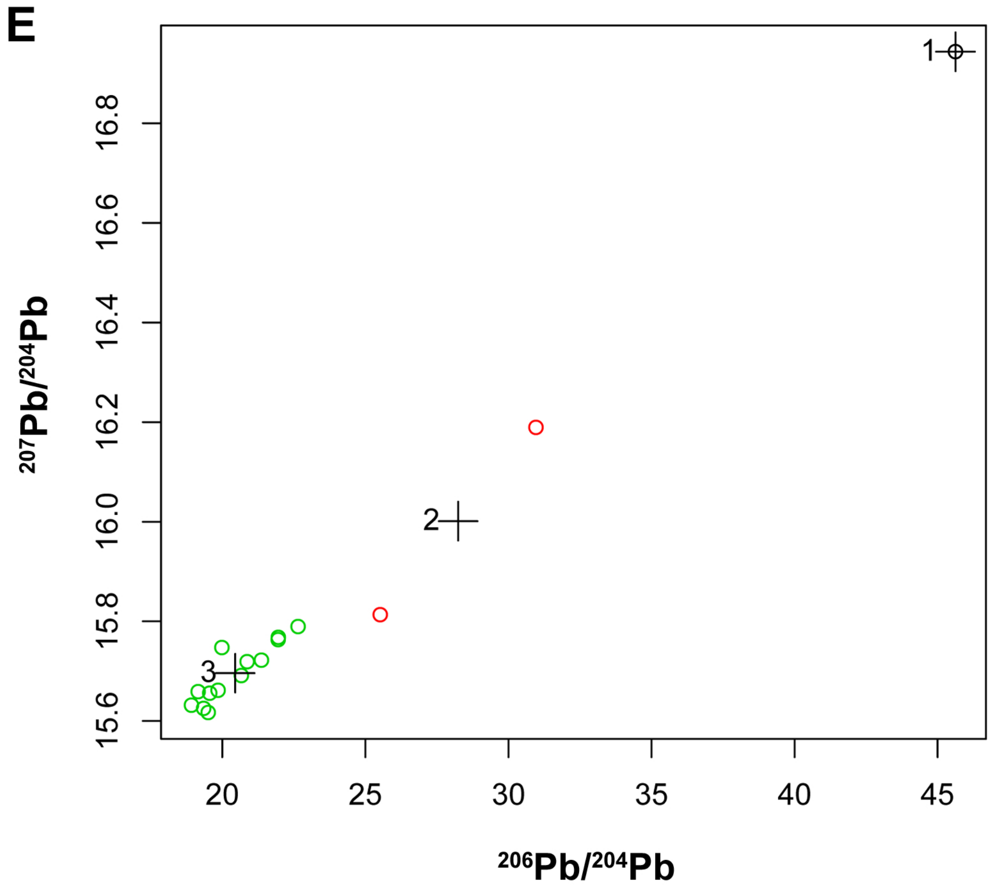
E
1
2
3
16.8
16.6
16.4
16.2
16.0
15.8
15.6
20
25
30
35
40
45
$^{207}Pb/^{204}Pb$
$^{206}Pb/^{204}Pb$

F

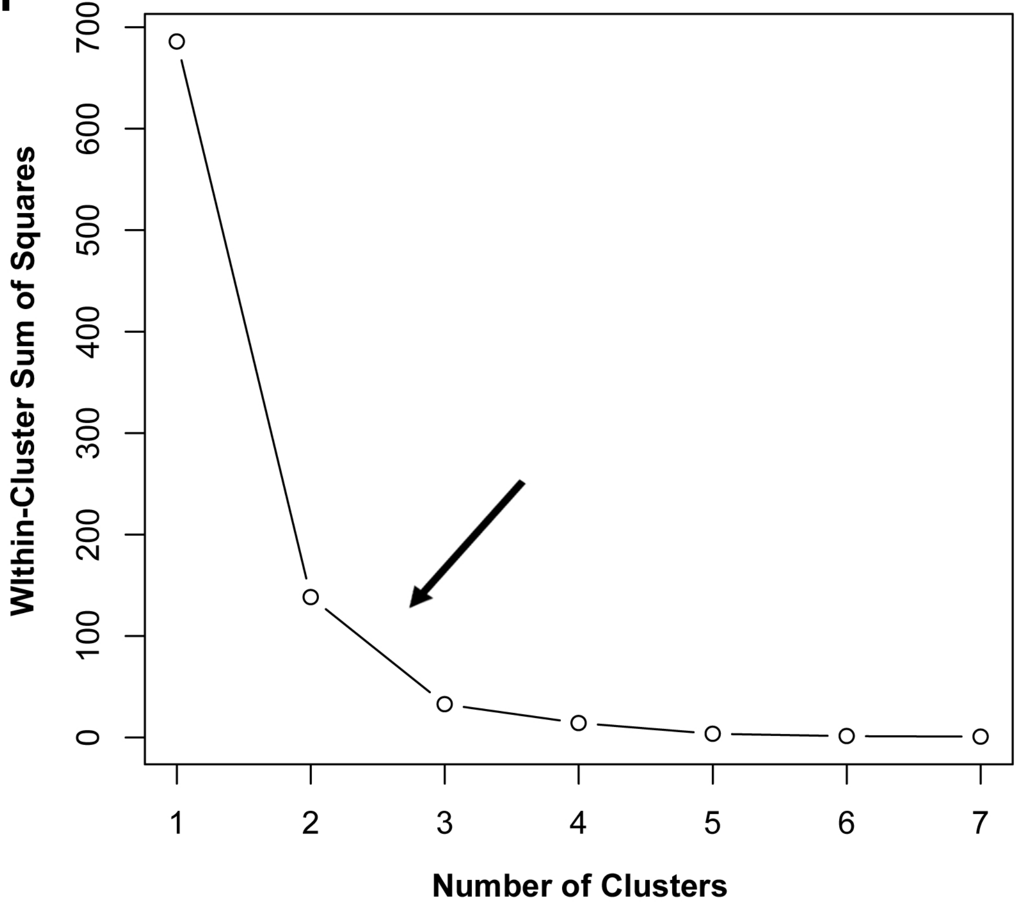

700
600
500
400
300
200
100
0
Within-Cluster Sum of Squares
1
2
3
4
5
6
7
Number of Clusters

G

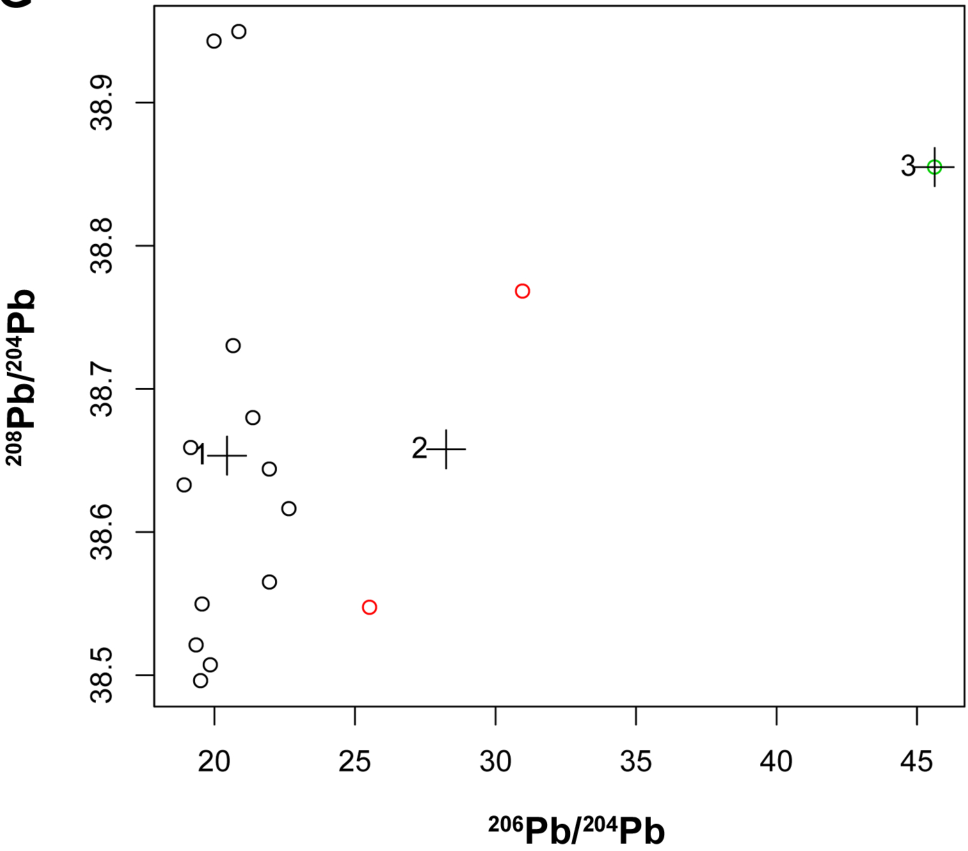
38.5
38.6
38.7
38.8
38.9
$^{208}Pb/^{204}Pb$
20
25
30
35
40
45
$^{206}Pb/^{204}Pb$
1
2
3

H

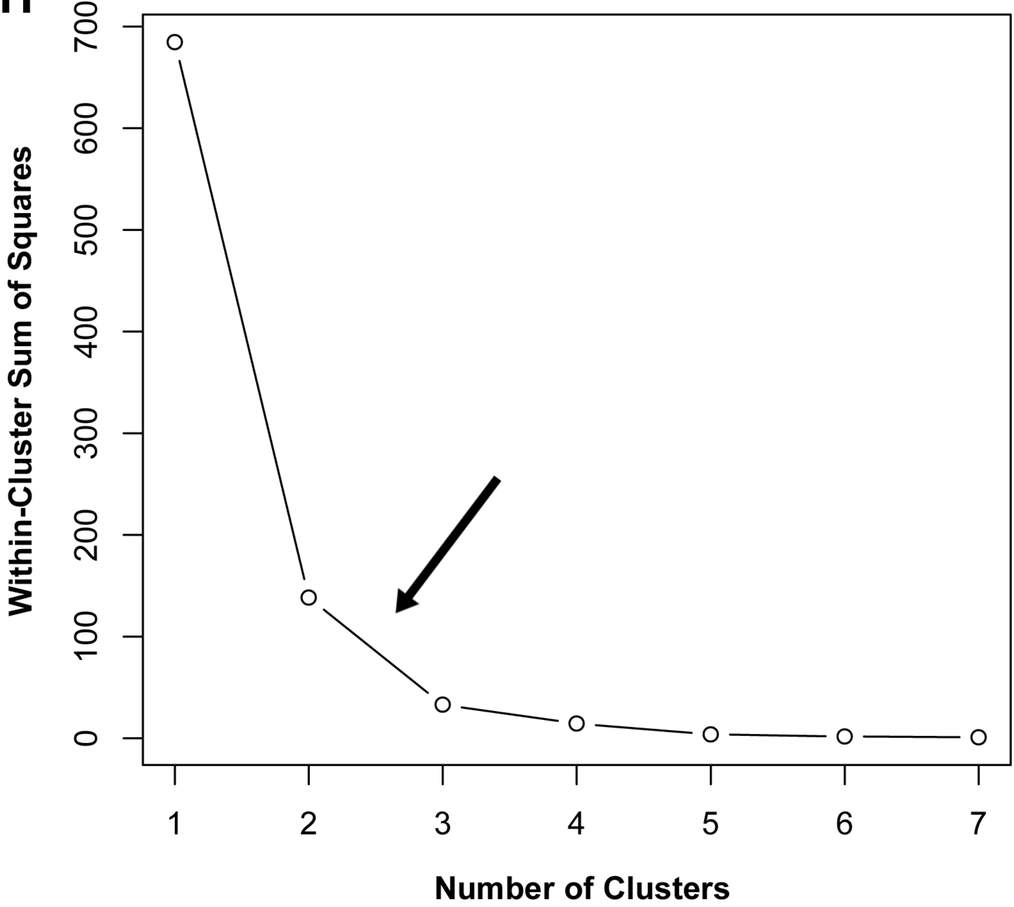

Within-Cluster Sum of Squares
0
100
200
300
400
500
600
700
1
2
3
4
5
6
7
Number of Clusters

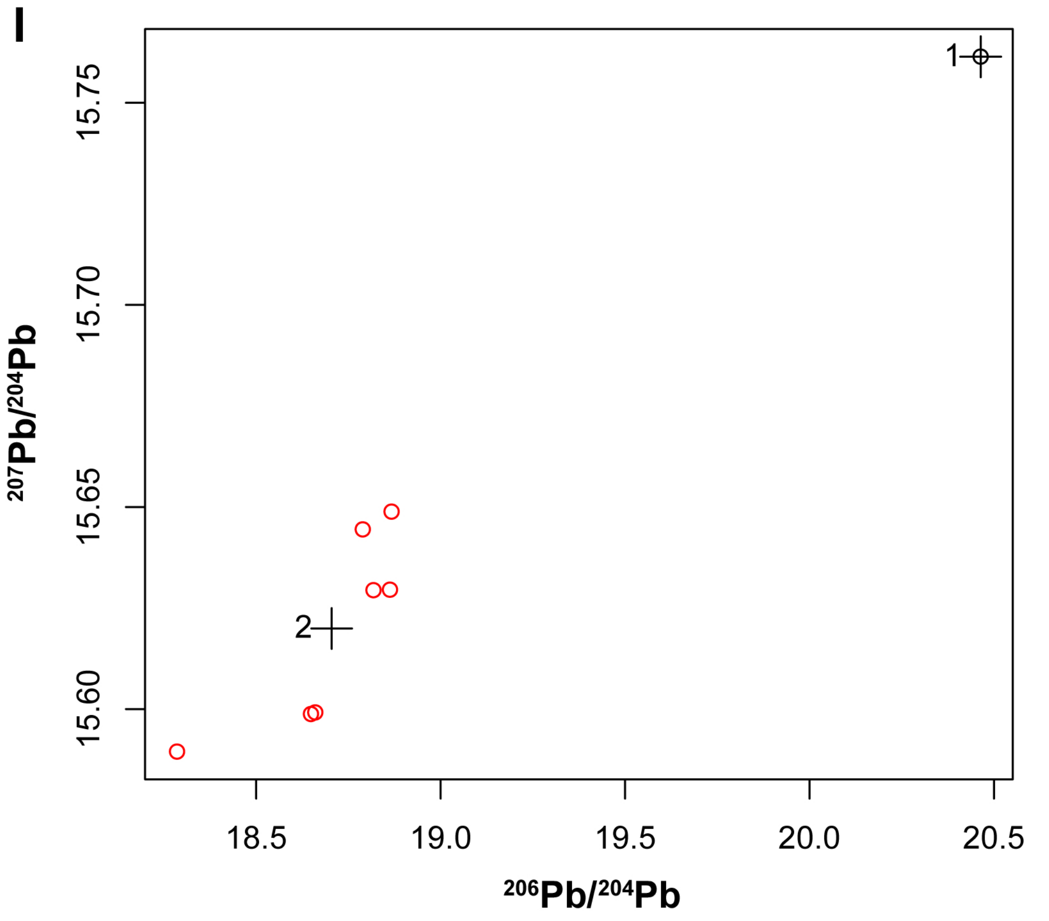

I
1
2
15.75
15.70
15.65
15.60
18.5
19.0
19.5
20.0
20.5
$^{207}Pb/^{204}Pb$
$^{206}Pb/^{204}Pb$

J

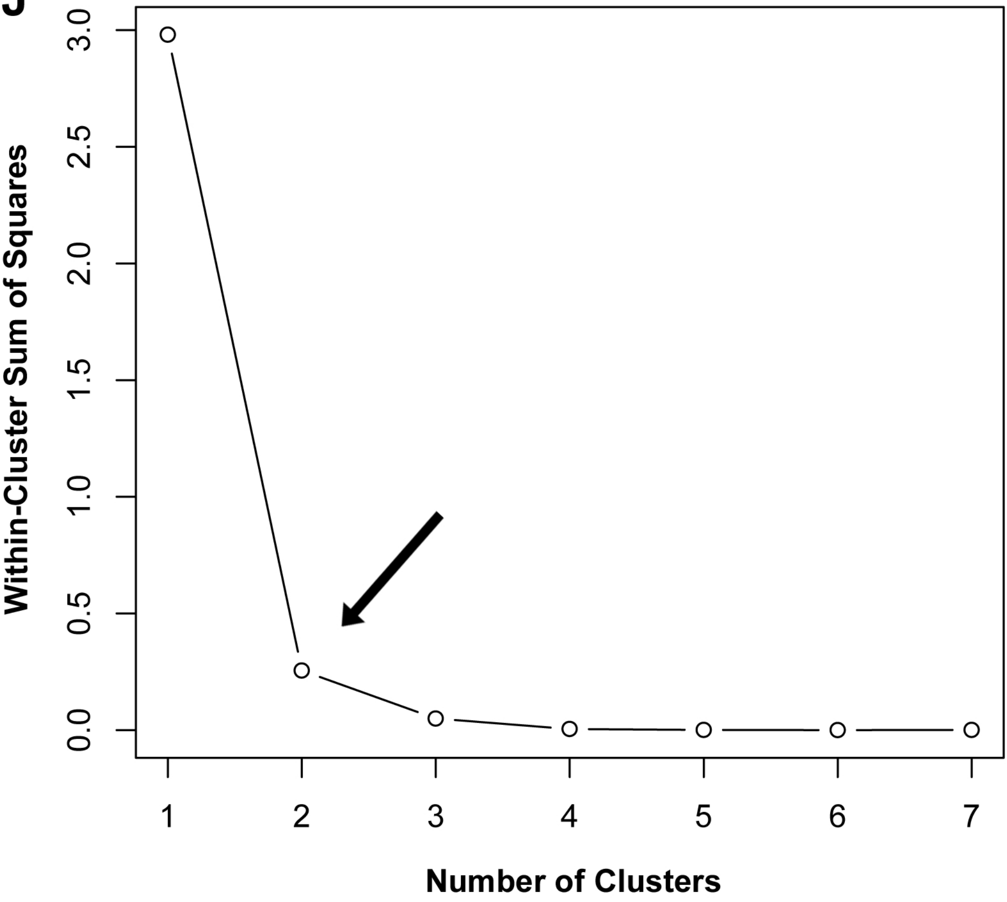
Within-Cluster Sum of Squares
3.0
2.5
2.0
1.5
1.0
0.5
0.0
1
2
3
4
5
6
7
Number of Clusters

K

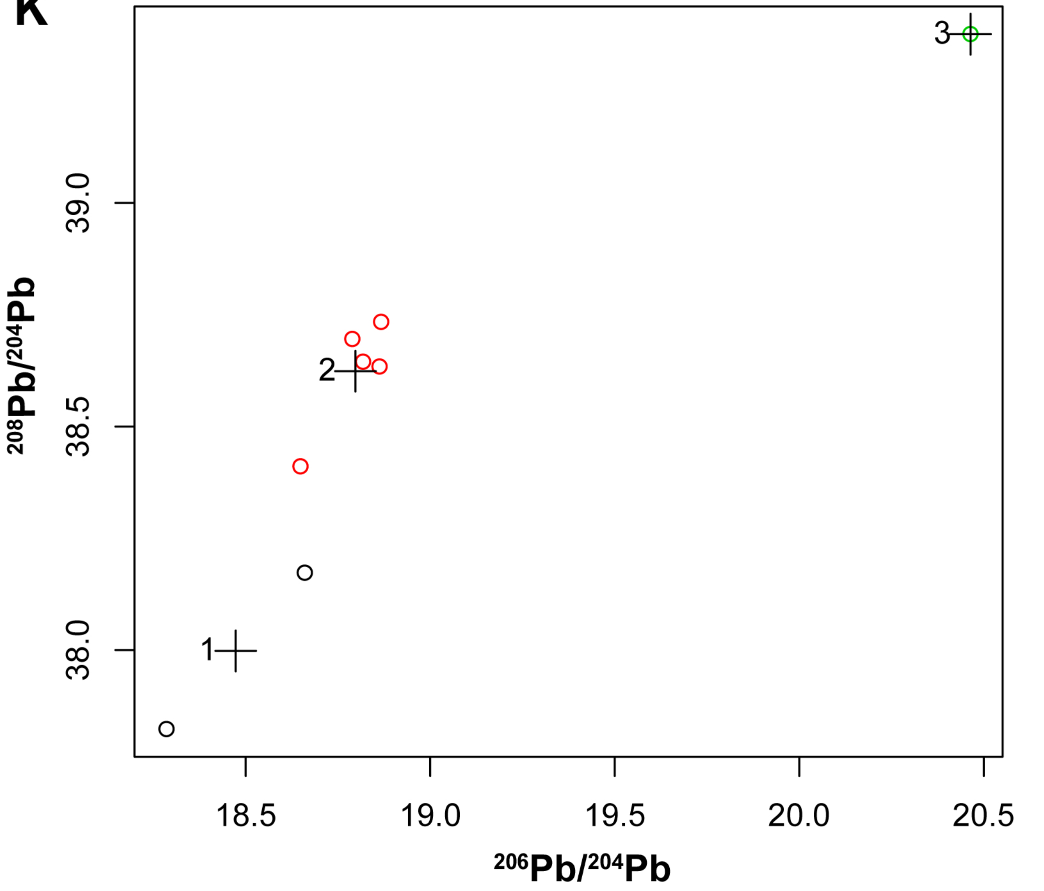

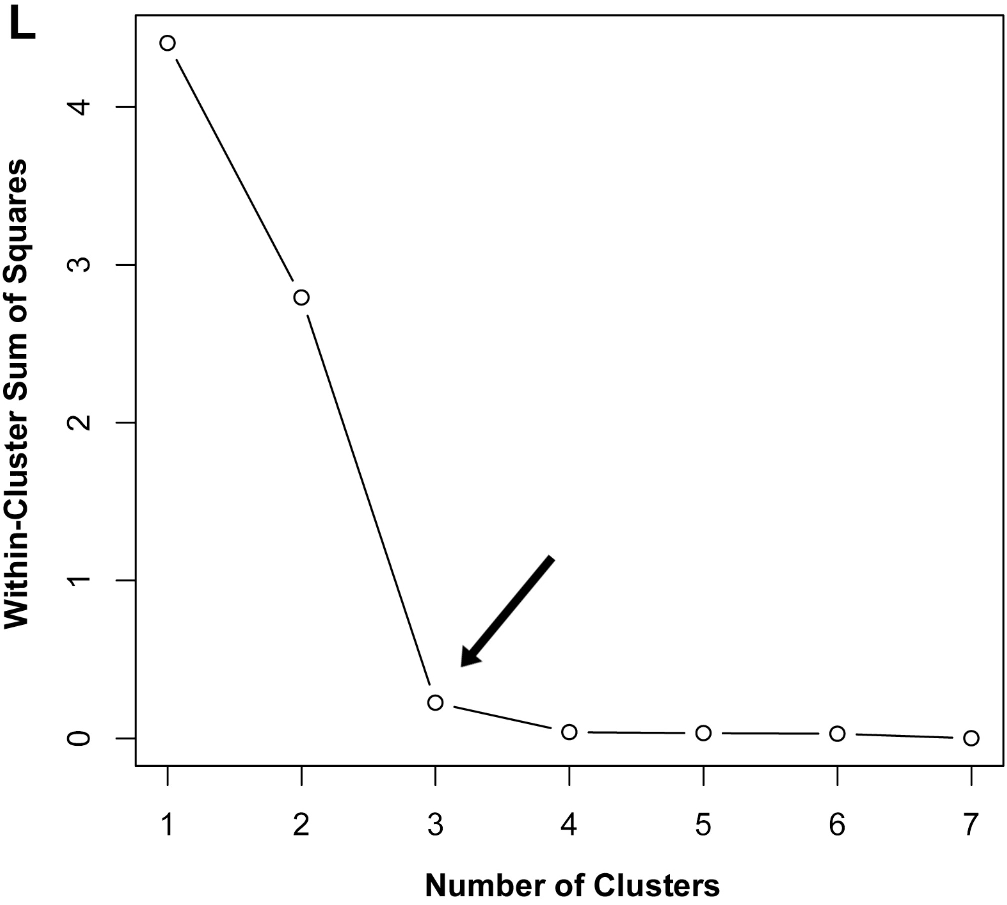
L
Within-Cluster Sum of Squares
0
1
2
3
4
1
2
3
4
5
6
7
Number of Clusters
